# Multimodal subspace identification for modeling discrete-continuous spiking and field potential population activity

**DOI:** 10.1088/1741-2552/ad1053

**Published:** 2024-03-01

**Authors:** Parima Ahmadipour, Omid G Sani, Bijan Pesaran, Maryam M Shanechi

**Affiliations:** 1 Ming Hsieh Department of Electrical and Computer Engineering, Viterbi School of Engineering, University of Southern California, Los Angeles, CA, United States of America; 2 Department of Neurosurgery, Perelman School of Medicine, University of Pennsylvania, Philadelphia, PA, United States of America; 3 Thomas Lord Department of Computer Science, Alfred E. Mann Department of Biomedical Engineering, and the Neuroscience Graduate Program, University of Southern California, Los Angeles, CA, United States of America

**Keywords:** dynamical systems, multimodal data, local field potentials, spiking activity, unsupervised learning, Poisson and Gaussian data

## Abstract

*Objective.* Learning dynamical latent state models for multimodal spiking and field potential activity can reveal their collective low-dimensional dynamics and enable better decoding of behavior through multimodal fusion. Toward this goal, developing unsupervised learning methods that are computationally efficient is important, especially for real-time learning applications such as brain–machine interfaces (BMIs). However, efficient learning remains elusive for multimodal spike-field data due to their heterogeneous discrete-continuous distributions and different timescales. *Approach.* Here, we develop a multiscale subspace identification (multiscale SID) algorithm that enables computationally efficient learning for modeling and dimensionality reduction for multimodal discrete-continuous spike-field data. We describe the spike-field activity as combined Poisson and Gaussian observations, for which we derive a new analytical SID method. Importantly, we also introduce a novel constrained optimization approach to learn valid noise statistics, which is critical for multimodal statistical inference of the latent state, neural activity, and behavior. We validate the method using numerical simulations and with spiking and local field potential population activity recorded during a naturalistic reach and grasp behavior. *Main results.* We find that multiscale SID accurately learned dynamical models of spike-field signals and extracted low-dimensional dynamics from these multimodal signals. Further, it fused multimodal information, thus better identifying the dynamical modes and predicting behavior compared to using a single modality. Finally, compared to existing multiscale expectation-maximization learning for Poisson–Gaussian observations, multiscale SID had a much lower training time while being better in identifying the dynamical modes and having a better or similar accuracy in predicting neural activity and behavior. *Significance.* Overall, multiscale SID is an accurate learning method that is particularly beneficial when efficient learning is of interest, such as for online adaptive BMIs to track non-stationary dynamics or for reducing offline training time in neuroscience investigations.

## 1. Introduction

Studies of neural population dynamics have mostly focused on a single modality of neural activity such as spikes or field potentials [1–29]. However, behaviors and internal states can be encoded across multiple neural modalities that measure different spatiotemporal scales [30–53], from small-scale spiking activity to field potentials which measure large-scale brain network activity [54–56]. For example, it has been shown that spiking and local field potential (LFP) activities exhibit shared dynamics, which are dominantly predictive of behavior during naturalistic reach-and-grasp movements [49]. Thus, building dynamical models that simultaneously incorporate multiple observation modalities is important for revealing how different spatiotemporal scales of neural population dynamics explain behavior. Further, such modeling can aggregate information across different spatiotemporal scales of neural activity to improve the performance of brain–machine interfaces (BMIs) [43, 44, 48–50]. We refer to dynamical modeling with multimodal observations as multiscale dynamical modeling.

Learning a multiscale dynamical model is challenging because different modalities have different statistical properties [43, 48, 49, 54–56]. For example, spike counts are discrete-valued action potential events with millisecond time-scales that are modeled well with Poisson distributions. In contrast, field potentials are continuous-valued and their spectral features evolve at slower time-scales than spikes, are extracted with slower time-steps, and are typically modeled with Gaussian distributions [41, 43, 44, 48, 49, 57–59]. To enable modeling of multimodal neural activity, we recently developed a multiscale expectation maximization (EM) method to learn a multiscale dynamical model [48, 49]. Similar to other EM methods [4, 60–62], this method aims to maximize the data log-likelihood iteratively but this time for joint Poisson–Gaussian data [48, 49]. However, EM is computationally expensive in terms of training given its iterative numerical learning approach, which can be burdensome or even prohibitive especially for real-time adaptive learning applications to track non-stationary dynamics, for example in closed-loop BMIs [63–68] (see Discussion). Thus, there is an important need for novel computationally efficient learning methods for multimodal neural data. Further, to enable real-time multiscale decoding/inference applications as multiscale EM [48, 49] does through the multiscale filter (MSF) [43], such novel methods should also produce models that support causal statistical inference from multimodal neural activity, which can be difficult to achieve (see Discussion).

Here, we develop an unsupervised learning method for multimodal Poisson–Gaussian data that is both computationally efficient in learning and enables causal multiscale inference to fuse information across data modalities during decoding [69]. The states in this model are latent and thus learning needs to be unsupervised with respect to these states. We also demonstrate the application of this method on multimodal spike-LFP neural activity recorded from the primate brain. To achieve computational efficiency in learning, we develop a novel analytical method for learning multiscale dynamical models. This method extends the subspace identification (SID) techniques which currently only support single modalities to multimodal data [23, 26, 29, 70–74]. Importantly, our method also introduces a novel approach for ensuring the validity of the learned noise statistics, which is critical for enabling statistical and causal inference of the latent states from multimodal data after learning is completed. We term this method multiscale SID. We emphasize that the multiscale SID method is distinct from multiscale EM [48, 49], which aims to iteratively maximize the log-likelihood and thus is computationally expensive given its iterative nature. Also, note that multiscale SID is an unsupervised learning method and thus distinct from multiscale filtering in prior work [43].

To date SID algorithms have been extended in various ways [23, 26, 29, 70–74], but not for addressing multiscale modeling. Traditional SID algorithms that model continuous signals operate by extracting the model parameters from empirically estimated cross-covariance matrices of future and past signals [19, 70, 71, 75]. SID has also been extended to two continuous signal sources to model the shared dynamics between continuous neural and behavioral signals [23], and for modeling the effect of input on neural-behavioral dynamics to dissociate input-driven and intrinsic neural dynamics [29]. Extensions of SID have also been developed for modeling discrete spike counts alone [72]. However, no SID method has been developed for joint dynamical modeling of multimodal observations that are a mix of continuous and discrete signals with different statistical properties.

To develop the multiscale SID method, we write a multiscale dynamical model with latent states and simultaneous discrete-continuous observations, e.g. consisting of spike counts and field potentials [48, 49]. We model the continuous observations as a linear Gaussian model of the latent states and the discrete spike counts as Poisson observations with a latent log firing rate that is a linear function of the same latent states. Extending the traditional SID to learn the parameters of this multiscale dynamical model involves several challenges.

The first challenge is related to the latent nature of the log firing rates [72, 76]. This latent nature means that the direct empirical estimation of the cross-covariance between the log firing rates and field potentials—which is needed by SID algorithms—is not possible. To address this challenge, we use statistical moment transformation [72, 77] and combine it with our multiscale dynamical model. In transforming statistical moments, a mathematical relationship between moments of two random variables is used to compute moments of one (which may lack direct observations) from the moments of the other, for which we have observations [72, 77]. Doing so, we find the multimodal cross-covariance between the latent log firing rates and the continuous modality indirectly by transforming the statistics that are directly computable from multimodal discrete-continuous observations.

The second challenge is to learn the model parameters while enforcing the learning of valid noise statistics. Learning valid noise statistics is not only important for accurate modeling, but also essential for statistical inference of latent states from neural observations, prediction of future neural activity, and neural decoding of behavior. Current covariance-based SID algorithms—i.e. those that can learn model parameters purely from data covariances as we do here—cannot guarantee learning of valid positive semidefinite (PSD) noise covariances [70–72]. In addition to valid PSD noise statistics, SID methods cannot ensure that noise statistics conform to the multiscale dynamical model structure for which an established causal inference algorithm exists, i.e. the multiscale filter [43]. Furthermore, the challenge of guaranteeing valid learned noise statistics remains unresolved even for the single-modal extension of SID for spike counts alone [72]. We address this challenge by devising a novel constrained optimization problem that revises the learned parameters of covariance-based SID methods to enforce valid noise statistics. We show that the model parameters learned by multiscale SID algorithm can then be used for causal multiscale inference of states, neural activity, and behavior.

Finally, multimodal observations may also be sampled at different rates, e.g. LFP spectral features often have a smaller sampling rate than binned spike counts [43, 48, 49], posing a challenge for jointly learning and describing their dynamics given these different timescales. We show that in our datasets, this challenge can be addressed via an interpolation approach in the training data during model learning, enabling multiscale SID to jointly learn the dynamics of both modalities, even if they are sampled at different rates. After model is learned, inference can be done without interpolation.

We validate the multiscale SID algorithm in numerical simulations and on motor cortical spike-LFP recordings of a non-human primate (NHP) performing a naturalistic three dimensional (3D) reach-and-grasp movement task [23, 49, 78]. We find that multiscale SID can accurately learn the multiscale dynamical model parameters. In addition, we find that combining spiking and field potential signals improves the identification accuracy of dynamical modes and prediction of behavior compared to using single-modal activity, showing that multiscale SID can accurately fuse information across modalities. We also compare the multiscale SID to the recent multiscale EM algorithm [48, 49] in terms of accuracy and the time it takes to compute the model parameters, i.e. the training time. In both simulations and the NHP dataset, we find that training time for multiscale EM was much higher than multiscale SID (about 180 times higher in simulations, and 30 times higher in the NHP dataset). Interestingly, this faster training time for multiscale SID did not lead to degradation of accuracy. Indeed, for some metrics such as dynamical mode identification and neural prediction in our NHP dataset multiscale SID outperformed multiscale EM and in other metrics they performed similarly.

Taken together, multiscale SID provides an accurate and efficient method for learning multiscale dynamical models for multimodal neural population data while also enabling causal statistical inference from multimodal data. These capabilities are especially important in real-time learning such as in closed-loop adaptive BMIs or closed-loop neuroscience experiments to track non-stationarity in neural representations [63–66, 68] (see Discussion).

## 2. Methods

**Figure 1.**
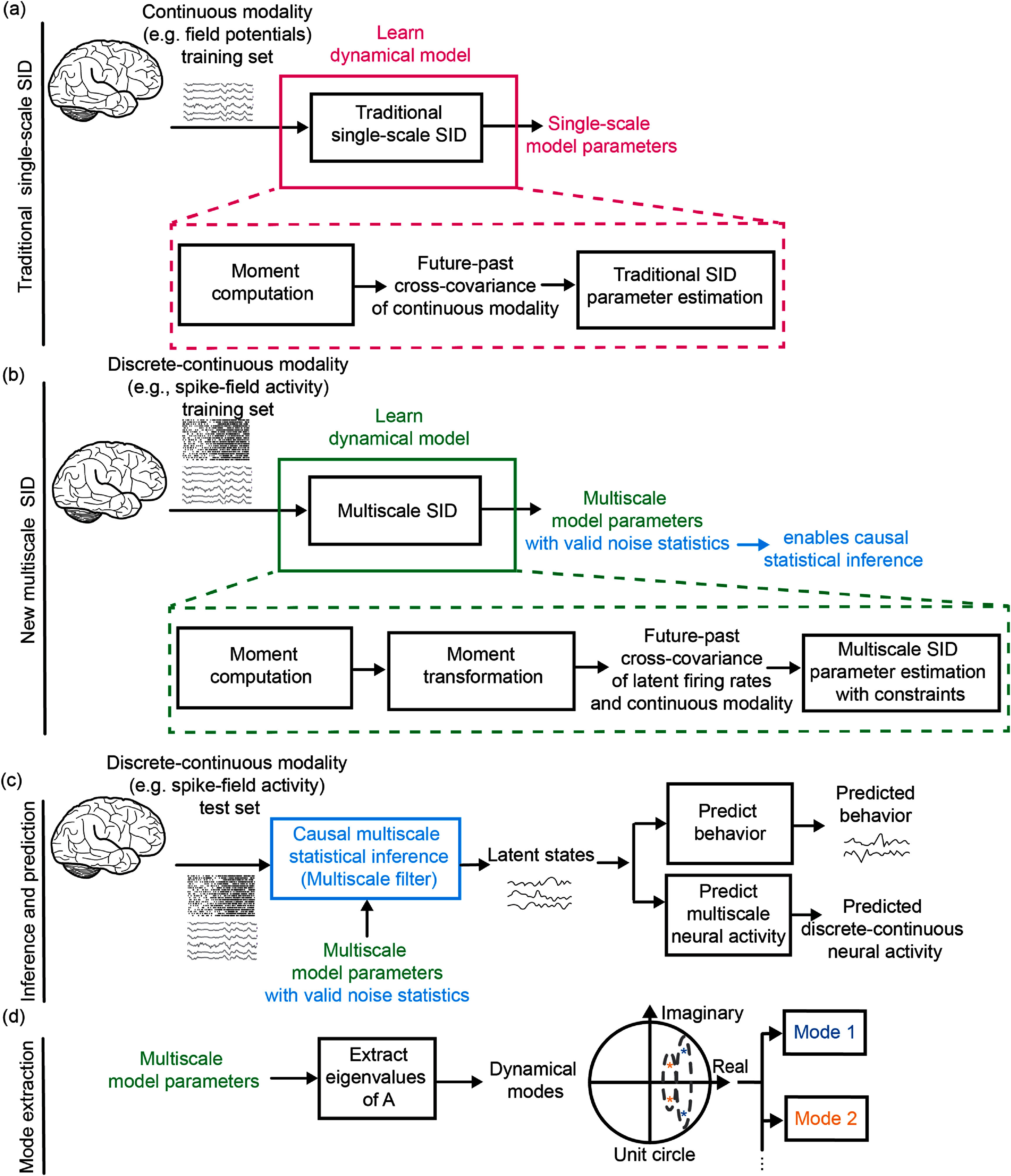
Multiscale SID algorithm for modeling and prediction of neural activity and behavior. (a) The traditional covariance-based SID algorithm learns the single-scale model parameters from a continuous modality (e.g. field potentials) in the training set (magenta box). These parameters are extracted from the future–past cross-covariance of the continuous modality **H**_*y*_, which can be computed directly from its observations. (b) The new multiscale SID algorithm (see algorithms 1 and 2) learns the multiscale model parameters from the discrete-continuous modalities (e.g. spike-field activity) in the training set (green box). Given that firing rates are latent, future–past cross-covariance of log firing rates and continuous modality **H**_*w*_ is not directly computable from the multimodal observations. Instead, we compute this cross-covariance **H**_*w*_ by transforming the moments of the discrete-continuous observations using the multiscale model equations. Then, we estimate the multiscale model parameters from **H**_*w*_ via SID methods. However, covariance-based SID methods even for a single modality do not guarantee valid noise statistics. We address this challenge by imposing added constraints in our SID method to enforce valid noise statistics within a novel optimization formulation. These constraints are critical for enabling multiscale statistical inference of latent states. (c) The learned valid parameter set is used to infer the latent states using a multiscale filter in the test set. These states are then used to predict behavior and the discrete-continuous neural activity using the learned model. (d) After learning the model parameters, the dynamical modes corresponding to a pair of complex conjugate eigenvalues or a real eigenvalue of **A** are computed.

In this section, we first provide a brief overview of multiscale SID in section 2.1 and a summary of the main steps of multiscale SID in algorithm 1 and figure 1. We then provide a detailed derivation of the algorithm as well as the details for all analyses in simulations and in the NHP data. Readers mainly interested in results can skip these sections after reading the brief method overview in section 2.1.

**Table:** 

**Algorithm 1.** Multiscale SID summary.
Summary of algorithm input and output:
**Input**: spiking activity $\{\mathbf{N}_1,\mathbf{N}_2,\mathbf{N}_3,{\ldots},\mathbf{N}_T\}$, continuous signals (e.g, field potential activity) $\{\mathbf{y}_1,\mathbf{y}_{M+1},\mathbf{y}_{2M+1}$ ${\ldots},\mathbf{y}_T\}$ and latent state dimension *n_x_*. Here, continuous signals may be available every *M* time steps.
**Output**: multiscale model parameter set $\mathcal{N} = \{\mathbf{A},\mathbf{C}_{y},\mathbf{C}_{z},\mathbf{Q},\mathbf{R}_{y},\mathbf{d}_z\}$.
1: Form the future–past spiking activity and future–past field potential activity vectors, i.e. $\mathbf{N}^\mathrm{fp}_t$ and $\mathbf{y}^\mathrm{fp}_t$, by stacking the time-lagged neural activity (equation (16)). Then, compute the empirical mean, covariance, and cross-covariance of these vectors directly from data. Note that to compute the above statistics, a linear interpolation is used when *M* > 1 to compute the missing samples of the continuous signal **y**_*t*_ at intermediate time steps; interpolation is however not needed during inference (see sections 2.2.2, 2.2.3 and 2.3.4).
2: Compute the following matrices/vectors by transforming the moments computed directly from data in step 1 according to the relations in equation set (18), per section 2.2.3 and appendix C:
(i) Cross-covariance of future and past concatenated latent firing rates and field potentials **H**_*w*_ (equation (13))
(ii) Covariance of concatenated latent log firing rates and field potentials Λ_0_
(iii) Mean of future–past log firing rate *µ* ^*z*^ (defined per equation (17))
3: Compute the singular value decomposition (SVD) of $\mathbf{H}_{w} = \mathbf{U}\mathbf{K}\mathbf{V}^{\prime}\cong \mathbf{U}_1\mathbf{K}_1\mathbf{V}_1^{\prime}$ and keep the top *n_x_* singular values.
4: Compute the estimates of extended observability matrix $\mathcal{O}_{w}$ and the extended reachability matrices $\mathcal{C}_{w}$ from **H**_*w*_ as $\mathcal{O}_{w} = \mathbf{U}_1\mathbf{K}_1^{\frac{1}{2}}$ and $\mathcal{C}_{w} = \mathbf{K}_1^{\frac{1}{2}}\mathbf{V}_1^{^{\prime}}$. $\mathcal{O}_{w}$ and $\mathcal{C}_{w}$ are also functions of model parameters **A**, **C**_*z*_, **C**_*y*_, $\mathbf{G}_{z}\triangleq \mathrm{Cov}[\mathbf{x}_{t+1},\mathbf{z}_{t}]$, $\mathbf{G}_{y}\triangleq\mathrm{Cov}[\mathbf{x}_{t+1},\mathbf{y}_{t}]$, per section 2.2.4 (equations (22) and (23)).
5: Read **C**_*z*_, **C**_*y*_, **G**_*z*_ and **G**_*y*_ from estimated $\mathcal{C}_{w}$ (equation (22)) and $\mathcal{O}_{w}$ (equation (23)) and estimate **A** from $\mathcal{O}_{w}$ using least squares.
6: Read **d**_*z*_ from the estimated *µ* ^*z*^ in step 2.
7: Compute state and observation noise covariances **Q** and **R**_*y*_ by solving a convex constrained optimization problem. This optimization problem enforces positive semidefinite **Q** and **R**_*y*_ and additional constraints to comply with the multiscale dynamical model and filter requirements, i.e. no noise for log firing rates and no correlation between state and field potential noises.

### 2.1. Overview of multiscale SID

We model the discrete-continuous spiking and field potential activity jointly as follows:

\begin{align*} \left\{ \begin{aligned} \mathbf{x}_{t+1} &amp; = \mathbf{A}\mathbf{x}_{t} + \mathbf{q}_{t},\\ \mathbf{y}_{t} &amp; = \mathbf{C}_{y}\mathbf{x}_{t} + \mathbf{r}_{y,t},\\ \mathbf{z}_{t} &amp; = \mathbf{C}_{z}\mathbf{x}_{t} + \mathbf{d}_{z},\\ \mathbf{N}_{t} &amp;\sim \mathrm{Poisson}\left[\mathrm{exp}\left(\mathbf{z}_{t}\right)\right]. \end{aligned} \right. \end{align*}

We refer to this state-space model as the multiscale dynamical model. Here the latent states $\mathbf{x}_t\in\mathbb{R}^{n_x}$ jointly describe the dynamics of continuous Gaussian signals denoted by $\mathbf{y}_t\in\mathbb{R}^{n_y}$ (e.g. field potentials) and discrete spike counts denoted by $\mathbf{N}_t\in\mathbb{R}^{n_z}$. The temporal structure, i.e. dynamics, of the latent state **x**_*t*_ is described using a linear state equation with the state transition matrix $\mathbf{A}\in\mathbb{R}^{n_x\times n_x}$. The continuous Gaussian signals **y**_*t*_ are modeled as a linear function of the latent states **x**_*t*_, and the discrete spike counts **N**_*t*_ are modeled as Poisson-distributed with latent log firing rate $\mathbf{z}_t\in\mathbb{R}^{n_z}$ [43, 48, 61, 72, 76, 79–81], which is a linear function of the same latent states. Also, $\mathbf{q}_t \in \mathbb{R}^{n_x}$ and $\mathbf{r}_{y,t}\in\mathbb{R}^{n_y}$ represent state and continuous observation noises and are modeled as uncorrelated white Gaussian noises with covariances $\mathbf{Q}\in\mathbb{R}^{n_x\times n_x}$ and $\mathbf{R}_y\in\mathbb{R}^{n_y\times n_x}$, respectively. Finally, $\mathbf{C}_y\in\mathbb{R}^{n_y\times n_x}$ and $\mathbf{C}_z\in\mathbb{R}^{n_z\times n_x}$ relate the continuous signal and the log firing rates to the latent state and **d**_*z*_ specifies the intercept (bias) of the log firing rate. The goal of multiscale SID is to learn all the multiscale model parameters $\mathcal{N} = \{\mathbf{A},\mathbf{C}_{y},\mathbf{C}_{z},\mathbf{Q},\mathbf{R}_{y},\mathbf{d}_z\}$ from multimodal neural training data.

We develop multiscale SID that resolves the learning challenges mentioned in the Introduction section and identifies the multiscale model parameter set $\mathcal{N} = \{\mathbf{A},\mathbf{C}_{y},\mathbf{C}_{z},\mathbf{Q},\mathbf{R}_{y},\mathbf{d}_z\}$ from multimodal neural observations **N**_*t*_ and **y**_*t*_ (figure 1(b)). Briefly, we resolve the challenge of estimating cross-covariances between latent log firing rates and observable field potentials—which is needed by SID—by finding the appropriate transformation of moments (see steps 1–2 of algorithm 2, section 2.2.3, [72]). We solve the challenge of estimating valid noise statistics and conforming to the multiscale model/inference structure through solving a constrained optimization problem with semidefinite programming (see step 9 of algorithm 2, sections 2.2.5–2.2.6, and equation (38), [82–84]). Finally, we address the challenge related to the different timescales of neural modalities by interpolating the slower modality (see step 1 of algorithm 2, section 2.2.3). Algorithm 1 provides a summary of the main steps of multiscale SID and algorithm 2 and section 2.2 provide more details.

**Table:** 

**Algorithm 2.** Multiscale SID.
Summary of algorithm input and output:
**Input**: discrete spiking observations $\{\mathbf{N}_1,\mathbf{N}_2,\mathbf{N}_3,{\ldots},\mathbf{N}_T\}$, continuous Gaussian observations (e.g. field potential activity) $\{\mathbf{y}_1,\mathbf{y}_{M+1},\mathbf{y}_{2M+1}{\ldots},\mathbf{y}_T\}$ and latent state dimension *n_x_*. Here, continuous Gaussian signals are available every *M* time steps.
**Output**: multiscale model parameter set $\mathcal{N} = \{\mathbf{A},\mathbf{C}_{y},\mathbf{C}_{z},\mathbf{Q},\mathbf{R}_{y},\mathbf{d}_z\}$.
1: Form the future–past spiking activity and future–past continuous modality activity vectors, i.e. $\mathbf{N}^\mathrm{fp}_t$ and $\mathbf{y}^\mathrm{fp}_t$ (equation (16)). Then, compute the following moments of these vectors directly from the training data (spiking and continuous modality activity): \begin{equation*} \left\{ \begin{aligned} \Sigma^{yy} &amp; \triangleq \mathrm{Cov}\left[\mathbf{y}_t^\mathrm{fp}\right], \quad \mu^{y} \,\,\,\triangleq \mathbb{E}\left[\mathbf{y}_t^\mathrm{fp}\right], \\[6pt] \Sigma^{NN} &amp; \triangleq \mathrm{Cov}\left[\mathbf{N}_t^\mathrm{fp}\right], \quad \mu^{N} \triangleq \mathbb{E}\left[\mathbf{N}_t^\mathrm{fp}\right], \\[6pt] \Sigma^{Ny} &amp; \triangleq \mathrm{Cov}\left[\mathbf{N}_t^\mathrm{fp},\mathbf{y}_t^\mathrm{fp}\right]. \\ \end{aligned} \right. \end{equation*} Note that you need to use interpolation to recover missing samples of **y**_*t*_ and be able to compute the covariances (see section 2.2.3).
2: Compute the future–past cross-covariance matrix $\mathbf{H}_w\triangleq\mathrm{Cov}[\mathbf{w}_t^\mathrm{f},\mathbf{w}_t^\mathrm{p}]$ by transforming the moments computed directly from data in the previous step according to the relations in equation set (18) and appendix C. $\mathbf{w}_t^\mathrm{f}$ ($\mathbf{w}_t^\mathrm{p}$) is defined by stacking the future (past) latent log firing rate vector and the future (past) continuous modality data vector (equations (14) and (15)).
3: Compute the SVD of $\mathbf{H}_{w} = \mathbf{U}\mathbf{K}\mathbf{V}^{\prime}\cong \mathbf{U}_1\mathbf{K}_1\mathbf{V}_1^{^{\prime}}$ and keep the *n_x_* largest singular values.
4: Compute the estimates of extended observability matrix $\mathcal{O}_{w}$ and the extended reachability matrix $\mathcal{C}_{w}$ (section 2.2.4) as $\mathcal{O}_{w} = (\begin{array}{c} \mathcal{O}_{z}\\ \mathcal{O}_{y} \end{array}) = \mathbf{U}_1\mathbf{K}_1^{\frac{1}{2}}$ and $\mathcal{C}_{w} = \left(\begin{array}{cc}\mathcal{C}_{z}&amp;\mathcal{C}_{y}\end{array}\right) = \mathbf{K}_1^{\frac{1}{2}}\mathbf{V}_1^{^{\prime}}$.
5: Read **C**_*z*_ and **C**_*y*_ from the submatrices of the extended observability matrix as $\mathbf{C}_z = \mathcal{O}_z(1:n_z,:)$ and $\mathbf{C}_y = \mathcal{O}_y(1:n_y,:)$, where : is the standard Matlab operation of selecting some or all elements from rows and columns.
6: Compute **A** as: \begin{equation*} A = \left( \begin{array}{c} \underline{\mathcal{O}_{z}}\\ \underline{\mathcal{O}_{y}} \end{array} \right)^{\dagger} \left( \begin{array}{c} \overline{\mathcal{O}_{z}}\\ \overline{\mathcal{O}_{y}} \end{array} \right). \end{equation*} Here $\underline{\mathcal{O}_{z}}$, $\underline{\mathcal{O}_{y}}$, $\overline{\mathcal{O}_{z}}$, and $\overline{\mathcal{O}_{y}}$ represent submatrices defined per equations (30)–(33) and, ${.}^{\dagger}$ represents pseudoinverse.
7: Read $\mathbf{G}_{z}\triangleq \mathrm{Cov}[\mathbf{x}_{t+1},\mathbf{z}_{t}]$ and $\mathbf{G}_{y}\triangleq\mathrm{Cov}[\mathbf{x}_{t+1},\mathbf{y}_{t}]$ from the submatrices of the extended reachability matrix as $\mathbf{G}_{z} = \mathcal{C}_{z}(:,1:n_z)$ and $\mathbf{G}_{y} = \mathcal{C}_{y}(:,1:n_y)$, respectively.
8: Compute covariance of stacked latent log firing rate and continuous observations, i.e. $\Lambda_{0}\triangleq\mathrm{Cov}[(\begin{array}{c}\mathbf{z}_{t}\\ \mathbf{y}_{t}\end{array})]$, and the mean of future–past log firing rate vector $\mu^{z} \triangleq \mathbb{E}[\mathbf{z}_t^\mathrm{fp}]$ (defined in section 2.2.3), again by transforming the moments computed in step 1 (relations are in equation set (18) and appendix C).
9: Compute state and observation noise covariances **Q** and **R**_*y*_ by solving the following optimization problem: \begin{equation*} \left\{ \begin{array}{l} \min_{\Sigma_{x}} \|\mathbf{S}\left(\Sigma_{x}\right)\|_\mathrm{F}+\|\mathbf{R}_{z}\left(\Sigma_{x}\right)\|_\mathrm{F}+\|\mathbf{R}_{zy}\left(\Sigma_{x}\right)\|_\mathrm{F}\\[5pt] \mbox{such that}~\Sigma_{x},~\mathbf{Q}\left(\Sigma_{x}\right),~\mathbf{R}_{y}\left(\Sigma_{x}\right)~ \mbox{are PSD.} \end{array} \right. \end{equation*} Here **Q**, **R**_*y*_, **R**_*z*_, $\mathbf{R}_{zy}$ and **S** (defined per equation set (2)) can be written in terms of the state covariance $\Sigma_{x}\triangleq\mathrm{Cov}(\mathbf{x}_{t})$ and estimated matrices in the previous steps, i.e. $\{\mathbf{C}_z,\mathbf{C_y},\mathbf{G}_z, \mathbf{G}_y,\mathbf{A},\Lambda_0\}$, according to equation set (39).
10: Read **d**_*z*_ from *µ* ^*z*^ as $\mathbf{d}_z = \mu^{z}(1:n_z)$.

Because the multiscale SID algorithm ensures valid noise statistics and conforms to the requirements of the multiscale dynamical model/inference structures, its learned parameters can readily enable statistical and causal inference of latent states from multimodal neural data. This can be done by incorporating the learned parameters inside an MSF [43]. Note that the same MSF can also use the parameters learned by multiscale EM [49], which is how we perform inference with multiscale EM as well. The inferred latent states can then be used to predict neural activity and behavior.

### 2.2. Detailed derivation of multiscale SID

It will be useful for our derivation of multiscale SID to rewrite the model from equation (1) in the following more general yet more compact form:

\begin{align*} \left\{ \begin{aligned} \mathbf{x}_{t+1} &amp; = \mathbf{A}\mathbf{x}_{t} + \mathbf{q}_{t},\\ \left( \begin{array}{c} \mathbf{z}_{t}\\ \mathbf{y}_{t} \end{array} \right) &amp; = \left( \begin{array}{c} \mathbf{C}_{z}\\ \mathbf{C}_{y} \end{array} \right) \mathbf{x}_{t} + \left( \begin{array}{c} \mathbf{r}_{z,t}\\ \mathbf{r}_{y,t} \end{array} \right) + \left( \begin{array}{c} \mathbf{d}_{z}\\ \mathbf{d}_{y} \end{array} \right),\\ \mathbf{N}_{t} &amp;\sim \mathrm{Poisson}\left[\mathrm{exp}\left(\mathbf{z}_{t}\right)\right]. \end{aligned} \right. \end{align*}

where $\mathbf{d}_{y}\in\mathbb{R}^{n_y} = 0$, $\mathbf{r}_{z,t}\in\mathbb{R}^{n_z} = 0$ and

\begin{align*} \mathrm{Cov}\left[\left(\begin{array}{c}\mathbf{q}_t\\\mathbf{r}_{z,t}\\ \mathbf{r}_{y,t}\end{array}\right)\right] = \left(\begin{array}{cc}\mathbf{Q} &amp; \mathbf{S} \\ \mathbf{S}^{^{\prime}} &amp; \mathbf{R} \end{array}\right), \end{align*}

with

\begin{align*} \mathbf{S} = \mathrm{Cov}\left[\mathbf{q}_t,\left(\begin{array}{c}\mathbf{r}_{z,t}\\ \mathbf{r}_{y,t}\end{array}\right)\right] = 0 \end{align*}

and

\begin{align*} \mathbf{R} = \mathrm{Cov}\left[\left(\begin{array}{c}\mathbf{r}_{z,t}\\ \mathbf{r}_{y,t}\end{array}\right)\right] = \left(\begin{array}{cc}\mathbf{R}_z &amp; \mathbf{R}_{zy}\\ \mathbf{R}_{zy}^{^{\prime}} &amp; \mathbf{R}_{y} \end{array}\right) = \left(\begin{array}{cc}0 &amp; 0\\ 0 &amp;\mathbf{R}_y \end{array}\right). \end{align*}

The multiscale dynamical model formulation used here is similar to that for developing the multiscale EM algorithm [48, 49]. It is a generalized linear model (GLM) and is amenable to efficient and tractable inference, e.g. with the MSF [43]. In prior work deriving and using the MSF [43, 48, 49], spikes were modeled as a binary point process, where there is either 0 or 1 spike in each time step ($1\unicode{x2A7E} N_t\unicode{x2A7E}0$). Nevertheless, the derivation of the MSF also holds for the case of a Poisson process, where there can be 0, 1 or more spikes in each time step ($N_t\unicode{x2A7E}0$), which we will use in this work.

In this section, we first review the traditional covariance-based SID algorithm used for modeling single-modal Gaussian observations [70, 71], such as field potentials (figure 1(a)). We then present the new multiscale SID algorithm for learning the multiscale dynamical model (figure 1(b)).

#### 2.2.1. The single-scale SID algorithm for Gaussian observations

We model the single-modal Gaussian observations, e.g. field potentials, using the first two lines of equation (1) that describe the temporal evolution of the latent state and the Gaussian observation as:

\begin{align*} \left\{ \begin{array}{l} \mathbf{x}_{t+1} = \mathbf{A}\mathbf{x}_{t} + \mathbf{q}_{t},\\ \,\,\,\,\,\,\,\,\mathbf{y}_{t} = \mathbf{C}_{y}\mathbf{x}_{t} + \mathbf{r}_{y,t},\\ \end{array} \right. \end{align*}

Briefly, the covariance-based SID algorithm finds the single-scale model parameters $\mathcal{M} = \{\mathbf{A}, \mathbf{C}_{y}, \mathbf{Q}, \mathbf{R}_{y}\}$ as follows [71] (figure 1(a)):

(i) Form the future and past observation vectors $\mathbf{y}_{t}^\mathrm{f}$ and $\mathbf{y}_{t}^\mathrm{p}$ by stacking time-lagged observations as:
  \begin{align*} \mathbf{y}_t^{\,\mathrm{f}}\triangleq\left( \begin{array}{c} \mathbf{y}_t \\ \mathbf{y}_{t+1} \\ \vdots\\ \mathbf{y}_{t+h_y-1} \\ \end{array} \right), \end{align*}
  \begin{align*} \mathbf{y}_t^\mathrm{p}\triangleq\left( \begin{array}{c} \mathbf{y}_{t-1} \\ \mathbf{y}_{t-2} \\ \vdots\\ \mathbf{y}_{t-h_y} \\ \end{array} \right). \end{align*}
  Here, *h_y_* is the horizon hyper-parameter of the SID algorithm, which needs to be specified by the user manually. The horizon *h_y_* must be larger than $\lceil\frac{n_x}{n_y}\rceil$ [70], such that the extended observability matrix $\mathcal{O}_{y}$ (equation (11)) obtained in the next step can have full column rank. This is a necessary condition for the final learned state-space model to be observable, meaning that the latent states can be uniquely estimated from the observations.
(ii) Empirically compute the future–past cross-covariance matrix **H**_*y*_ from the data formed in step (i) as $\mathbf{H}_{y}\triangleq\mathrm{Cov}\left[\mathbf{y}_t^\mathrm{f},\mathbf{y}_t^\mathrm{p}\right]$. It is easy to see that **H**_*y*_ can be written in terms of auto covariances of **y**_*t*_ at different time delays *k*, i.e. in terms of $\Lambda_{k}^{yy}\triangleq\mathrm{Cov}[\mathbf{y}_{t+k},\mathbf{y}_{t}]$, assuming stationary auto covariances across time:
  \begin{align*} \mathbf{H}_{y} = \left( \begin{array}{cccc} \Lambda_{1}^{yy} &amp; \Lambda_{2}^{yy} &amp; \cdots &amp; \Lambda_{h_y}^{yy} \\[2pt] \Lambda_{2}^{yy} &amp; \Lambda_{3}^{yy} &amp; \cdots &amp; \Lambda_{h_y+1}^{yy} \\[2pt] \vdots &amp; \vdots &amp; \vdots &amp; \vdots \\[2pt] \Lambda_{h_y}^{yy} &amp; \Lambda_{h_y+1}^{yy} &amp; \cdots &amp; \Lambda_{2h_y-1}^{yy} \\ \end{array} \right). \end{align*}
  Since $\Lambda_{k}^{yy} = \mathbf{C}_y\mathbf{A}^{k-1}\mathbf{G}_y$ with $\mathbf{G}_y\triangleq\mathrm{Cov}[\mathbf{x}_{t+1},\mathbf{y}_t]$ (see appendix D), **H**_*y*_ can be decomposed in terms of **A**, **C**_*y*_ and **G**_*y*_, as:
  \begin{equation*} {\mathbf{H}_y = \mathcal{O}_{y}\mathcal{C}_{y}} \end{equation*}
  \begin{equation*} {\mathcal{O}_{y} = \left(\begin{array}{c} \mathbf{C}_{y} \\ \mathbf{C}_{y}\mathbf{A} \\ \vdots \\ \mathbf{C}_{y}\mathbf{A}^{h_y-1} \\ \end{array} \right)} \end{equation*}
  \begin{equation*} {\mathcal{C}_{y} = \left(\begin{array}{cccc} \mathbf{G}_{y} &amp; \mathbf{A}\mathbf{G} _{y} &amp; \cdots &amp; \mathbf{A}^{\left(h_y-1\right)}\mathbf{G}_{y} \end{array} \right)} \end{equation*}
  where $\mathcal{O}_y$ and $\mathcal{G}_y$ are termed extended observability matrix and extended reachability matrix, respectively [70, 71].
(iii) Take singular value decomposition (SVD) of empirically estimated **H**_*y*_ from step (ii) to decompose it into the extended observability ($\mathcal{O}_y$) and reachability ($\mathcal{G}_y$) matrices (see [70, 71] for details). As shown in the previous step, these estimated $\mathcal{O}_y$ and $\mathcal{G}_y$ matrices will be functions of the yet-unknown model parameters **A**, **C**_*y*_ and **G**_*y*_.
(iv) Find model parameters $\mathcal{M} = \{\mathbf{A}, \mathbf{C}_{y}, \mathbf{Q}, \mathbf{R}_{y}\}$ from estimates of $\mathcal{O}_y$ and $\mathcal{G}_y$ and by solving a linear least squares problem and an algebraic Riccati equation. Interested readers can refer to [70, 71] for more details.

These steps conclude the traditional covariance-based SID algorithm for learning single-scale models with Gaussian observations.

#### 2.2.2. Outstanding challenges for developing a multiscale SID

The traditional covariance-based SID (reviewed in section 2.2.1) is not applicable to the multiscale model from equation (1) due to three standing challenges. In this section, we will explain these challenges for developing a multiscale SID and provide a brief explanation of our approach for addressing them in this section.

The first challenge in developing multiscale SID is that for the spiking modality, the log firing rates denoted by **z**_*t*_ are not observed. Rather, only a stochastic Poisson-distributed spike count time series, denoted by **N**_*t*_, is observed, which is nonlinearly related to the log-firing rates (equation (1)). Thus, one cannot directly compute the empirical statistics of log firing rates as is possible with the Gaussian continuous modality **y**_*t*_. For example, prior work for single-modal spiking activity has computed the covariance of unobservable log firing rates by transforming the moments of the observable spike counts [72] through their computable relationship. In our multiscale dynamical model, in addition to the auto covariances at different time delays for each modality on its own, the cross-covariance terms between the two modalities at different time delays are required for parameter learning. However, since spiking activity is one of the modalities, we cannot directly estimate the cross-covariance of its log firing rates with the continuous modality (**y**_*t*_, e.g. field potentials). To infer these cross-covariance terms at different time delays, we use the method of moment transformation similar to prior work on single-modal spiking activity [72], but this time we find the transformation for the cross-covariance between discrete and continuous multimodal observations (section 2.2.3). Specifically, because the relationship between the moments of discrete spikes and their associated log firing rates is analytically derivable as we show in equation (18), we can transform the computable moments between observable spikes and field potentials to find the moments between the latent/unobservable log firing rates and field potentials. Note that prior work [72] did not address the modeling of multimodal Poisson observations simultaneously with Gaussian observations—which also necessitates modeling of their joint statistics (equation (18))—, nor did it address the other two standing challenges in multiscale SID, which we outline next. Also, note that after identifying the multiscale model parameters using multiscale SID, one can infer the instantaneous log firing rates if desired using an MSF [43] with the identified parameters.

The second challenge in developing multiscale SID is related to ensuring that learned noise statistics are valid and conform to the multiscale model structure in equation set (2) and the assumptions of its latent state inference algorithm, i.e. the MSF [43, 48]. Covariance-based SID methods [71], including the single-scale SID with Poisson observations [72], do not guarantee the validity of their learned noise covariance parameters. For example, these methods may learn noise covariance matrices **Q** or **R**_*y*_ that are not PSD, which is a necessary condition for a model representing real-valued time series and for enabling the statistical inference of its latent states. We address this challenge by devising a convex constrained optimization problem that finds a valid set of noise statistics for the model (section 2.2.6).

The third challenge in developing multiscale SID is that Gaussian continuous observations such as field potentials **y**_*t*_ and spike count observations **N**_*t*_ may be available at different timescales due to differences in their sampling rate. This means that some timesteps may have the slower modality as missing observations. We address this challenge by resampling and interpolating the slower signals within the training data and prior to forming the noise covariances, which can work when the Nyquist sampling rate criterion is met for the slower signal as is often the case for field potential signals (section 2.2.3). Once model parameters are learned, we no longer need to perform interpolation in the test set; instead, we will estimate the latent states and predict the neural activity using the MSF [43], which can process multimodal data with different sampling rates.

In the following sections, we provide details of how we address each of these challenges and finally estimate all the model parameters $\mathcal{N} = \{\mathbf{A},\mathbf{C}_{y},\mathbf{C}_{z},\mathbf{Q},\mathbf{R}_{y},\mathbf{d}_z\}$.

#### 2.2.3. Empirical estimation of the future–past cross-covariances between the log firing rates and the continuous modality (i.e. **H**_*w*_)

To model multimodal data per equation (1) (figure 1(b)), we first empirically compute the following future–past cross-covariance matrix **H**_*w*_ and subsequently estimate model parameters from it:

\begin{align*} \mathbf{H}_{w} \triangleq \mathrm{Cov}\left[\mathbf{w}_t^\mathrm{f},\mathbf{w}_t^\mathrm{p}\right] \end{align*}

with

\begin{align*} \mathbf{w}^\mathrm{f}_{t}\triangleq \left(\begin{array}{c}\mathbf{z}^\mathrm{f}_{t}\\ \hline\mathbf{y}^\mathrm{f}_{t}\end{array}\right)\triangleq \left( \begin{array}{c} \mathbf{z}_t \\ \mathbf{z}_{t+1} \\ \vdots\\ \mathbf{z}_{t+h_z-1} \\ \hline \mathbf{y}_t \\ \mathbf{y}_{t+1} \\ \vdots\\ \mathbf{y}_{t+h_y-1} \\ \end{array} \right), \end{align*}

\begin{align*} \mathbf{w}_t^\mathrm{p}\triangleq \left(\begin{array}{c}\mathbf{z}^\mathrm{p}_{t}\\ \hline\mathbf{y}^\mathrm{p}_{t}\end{array}\right)\triangleq \left( \begin{array}{c} \mathbf{z}_{t-1} \\ \mathbf{z}_{t-2} \\ \vdots\\ \mathbf{z}_{t-h_z} \\ \hline \mathbf{y}_{t-1} \\ \mathbf{y}_{t-2} \\ \vdots\\ \mathbf{y}_{t-h_y} \\ \end{array} \right). \end{align*}

Here $\mathbf{w}_{t}^\mathrm{f}$ ($\mathbf{w}_{t}^\mathrm{p}$) is formed by stacking the future (past) latent log firing rate vector and the future (past) observed continuous modality vector. *h_z_* is another horizon parameter that needs to be selected manually similar to *h_y_* (section 2.2.1) and corresponds to spiking activity. In all analyses in this work, we set horizon parameters as $h_y = h_z = 10$ unless otherwise stated.

Note that log firing rates **z**_*t*_ are not directly observed, and thus **H**_*w*_ (equations (13)–(15)) cannot be directly estimated from data as a sample covariance. To estimate **H**_*w*_ from data, we need estimates of auto covariances of **y**_*t*_ as well as auto covariances of **z**_*t*_ and cross-covariances of **y**_*t*_ and **z**_*t*_ at different time delays. While we can directly estimate moments of **y**_*t*_ from the continuous observations, we cannot do the same for auto covariances of **z**_*t*_ and cross-covariances of **y**_*t*_ and **z**_*t*_ as log firing rate **z**_*t*_ is not observable. However, we can use the fact that the model in equation (1) dictates a computable relationship between moments of **y**_*t*_ and **z**_*t*_ and those of **y**_*t*_ and **N**_*t*_, which can be directly estimated from the discrete-continuous observations. So we transform moments [72] that are directly computable from data (i.e. moments of **y**_*t*_ and **N**_*t*_ and their cross-terms) to the unknown moments required to estimate **H**_*w*_, i.e. moments of **y**_*t*_ and **z**_*t*_ and their cross-terms, i.e. the cross-terms between the two modalities.

To do so, first we define the future–past vector of the continuous modality activity $\mathbf{y}^\mathrm{fp}_{t}\in \mathbb{R}^{2h_yn_y}$ by stacking $\mathbf{y}^\mathrm{f}_{t}$ and $\mathbf{y}^\mathrm{p}_{t}$ as:

\begin{align*} \mathbf{y}^\mathrm{fp}_{t}\triangleq \left(\begin{array}{c}\mathbf{y}^\mathrm{f}_{t}\\ \hline\mathbf{y}^\mathrm{p}_{t}\end{array}\right)\triangleq \left( \begin{array}{c} \mathbf{y}_t \\ \mathbf{y}_{t+1} \\ \vdots\\ \mathbf{y}_{t+h_y-1} \\ \hline \mathbf{y}_{t-1} \\ \mathbf{y}_{t-2} \\ \vdots\\ \mathbf{y}_{t-h_y} \\ \end{array} \right). \end{align*}

Similarly we define $\mathbf{N}^\mathrm{fp}_{t}$, $\mathbf{z}^\mathrm{fp}_{t}$ for variables **N**_*t*_ and **z**_*t*_ by stacking the corresponding future and past vectors. Then we define the mean denoted by *µ*, and the auto-covariance and cross-covariance denoted by Σ of these variables as follows:

\begin{align*} \left\{ \begin{aligned} \Sigma^{yy}&amp;\triangleq\mathrm{Cov}\left[\mathbf{y}_t^\mathrm{fp}\right],\qquad\qquad\mu^{y} \triangleq\mathbb{E}\left[\mathbf{y}_t^\mathrm{fp}\right], \checkmark\\[6pt] \Sigma^{NN}&amp;\triangleq\mathrm{Cov}\left[\mathbf{N}_t^\mathrm{fp}\right],\quad\qquad\,\,\,\mu^{N}\triangleq\mathbb{E}\left[\mathbf{N}_t^\mathrm{fp}\right],\checkmark\\[6pt] \Sigma^{Ny}&amp;\triangleq\mathrm{Cov}\left[\mathbf{N}_t^\mathrm{fp},\mathbf{y}_t^\mathrm{fp}\right],\qquad \qquad \qquad\,\,\,\quad \checkmark\\[6pt] \Sigma^{zz}&amp;\triangleq\mathrm{Cov}\left[\mathbf{z}_t^\mathrm{fp}\right],\qquad\qquad\mu^{z}\triangleq\mathbb{E}\left[\mathbf{z}_t^\mathrm{fp}\right],\times\\[6pt] \Sigma^{zy}&amp;\triangleq\mathrm{Cov}\left[\mathbf{z}_t^\mathrm{fp},\mathbf{y}_t^\mathrm{fp}\right],\qquad\qquad\qquad\,\,\,\,\,\,\quad\times\\ \end{aligned} \right.. \end{align*}

We have empirical estimates of $\Sigma^{yy}$ and *µ* ^*y*^ directly from the continuous observations, $\Sigma^{NN}$ and *µ* ^*N*^ directly from spike observations and $\Sigma^{Ny}$ directly from both observations (first three lines in equation set (17). These empirical moments correspond to the output of moment computation block in figure 1(b). Appendix A explains the empirical computation of these moments. We then compute $\Sigma^{zy}$, $\Sigma^{zz}$ and *µ* ^*z*^ (last two lines in equation set (17)) that are not directly computable from observations, by a moment transformation procedure based on the following relations:

\begin{align*} \left\{ \begin{aligned} \mu^{z}_{i} &amp; = 2\text{log}\left(\mu^{N}_{i}\right)-\frac{1}{2}\text{log}\left(\Sigma^{NN}_{i,i}+\left(\mu^{N}_{i}\right)^{2}-\mu^{N}_{i}\right),\\[5pt] \Sigma^{zz}_{i,i} &amp; = \text{log}\left(\Sigma^{NN}_{i,i}+\left(\mu^{N}_{i}\right)^{2}-\mu^{N}_{i}\right)-\text{log}\left(\left(\mu^{N}_{i}\right)^{2}\right),\\[5pt] \Sigma^{zz}_{i,j} &amp; = \text{log}\left(\Sigma^{NN}_{i,j}+\mu^{N}_{i}\mu^{N}_{j}\right)-\text{log}\left(\mu^{N}_{i}\mu^{N}_{j}\right),\\[5pt] \Sigma^{zy}_{i,j} &amp; = \Sigma^{Ny}_{i,j}/\mu^{N}_{i}\\ \end{aligned} \right. \end{align*}

where ._*i*_ refers to the *i*th element of a vector, and $._{i,j}$ to the element in the *i*th row and *j*th column of a matrix. In appendix B, we derive the relation for computing $\Sigma^{zy}$ in equation set (18), which is the cross-covariance between the two discrete-continuous modalities. The relations for computing *µ* ^*z*^ and $\Sigma^{zz}$ in equation set (18) are derived in [72] where single-scale SID is derived for a single modality with Poisson distribution.

The above procedure addresses the first challenge in developing multiscale SID (see section 2.2.2), giving an estimate of the future–past cross-covariance matrix **H**_*w*_ (figure 1(b)). See appendix C for constructing **H**_*w*_ based on equations (17) and (18). We also compute $\Lambda_{0}\triangleq\mathrm{Cov}[(\begin{array}{c}\mathbf{z}_{t}\\ \mathbf{y}_{t}\end{array})]$ from the quantities computed in equation (17) (appendix C), to be used when we subsequently estimate all the model parameters $\mathcal{N} = \{\mathbf{A},\mathbf{C}_{y},\mathbf{C}_{z},\mathbf{Q},\mathbf{R}_{y},\mathbf{d}_z\}$ using the estimated **H**_*w*_, Λ_0_ and *µ* ^*z*^ later in section 2.2.5.

To address the challenge of potentially different sampling rates in developing multiscale SID (see section 2.2.2), we proceed as follows. For empirical estimation of $\Sigma^{yy}$ and $\Sigma^{Ny}$ from the discrete-continuous observations in the training data, we first interpolate the slower modality to make the sampling rate of the two modalities the same. Here, we assume that we observe the spiking activity at every time step, while we may compute the continuous modality such as field potential power features every $M\unicode{x2A7E}1$ time steps (section 2.1) and therefore have continuous observations **y**_*t*_ every $M\unicode{x2A7E}1$ time steps. This is often the case in neural spike-field datasets [43, 49, 56]. Thus, we increase the sampling rate of the continuous observations by a factor of *M* by filling in their missing samples with zeros and then applying a zero-phase finite impulse response (FIR) filter (see [85], we use the ‘interp’ command in MATLAB).

It is worth noting that by performing interpolation for the slower modality, we assume that the multiscale data is collected at an appropriate sampling rate for each modality, such that information has not already been irreversibly lost due to aliasing when the data was originally sampled [86]—i.e. we assume that Nyquist sampling rate requirements are met. This assumption is reasonable because any information lost due to aliasing is not retrievable by any learning method and thus interpolation to recover the existing information is a reasonable approach. Moreover, note that this interpolation is only needed in the training data for computations of equation (17) when learning the model parameters and not for prediction of neural activity or behavior after the model parameters are learned.

We address the challenge of ensuring valid noise statistics in sections 2.2.5 and 2.2.6 after first presenting how model parameters relate to the computed covariances.

#### 2.2.4. Relation of **H**_*w*_ to the model parameters

In this section, we will show how **H**_*w*_ (equation (13)) can be written in terms of the multiscale model parameters, which we will later use to extract the model parameters from **H**_*w*_ in section 2.2.5. As discussed in section 2.2.3, we can write **H**_*w*_ in terms of the cross and auto covariances of **y**_*t*_ and **z**_*t*_ at different time delays *k*, i.e. in terms of $\Lambda_{k}^{yy}\triangleq\mathrm{Cov}[\mathbf{y}_{t+k}, \mathbf{y}_{t}]$, $\Lambda_{k}^{zz}\triangleq\mathrm{Cov}[\mathbf{z}_{t+k}, \mathbf{z}_{t}]$, $\Lambda_{k}^{yz}\triangleq\mathrm{Cov}[\mathbf{y}_{t+k}, \mathbf{z}_{t}]$ and $\Lambda_{k}^{zy}\triangleq\mathrm{Cov}[\mathbf{z}_{t+k}, \mathbf{y}_{t}]$ as:

\begin{align*} \mathbf{H}_{w} &amp; = \mathrm{Cov}\left[\mathbf{w}_t^\mathrm{f},\mathbf{w}_t^\mathrm{p}\right] = \left(\begin{array}{c|c} \mathbf{H}_{w}^{zz}&amp; \mathbf{H}_{w}^{zy}\nonumber\\ \hline \mathbf{H}_{w}^{yz}&amp; \mathbf{H}_{w}^{yy} \end{array}\right) =\nonumber\\&amp;\quad \left( \begin{array}{ccc|ccc} \Lambda_{1}^{zz} &amp; \cdots &amp; \Lambda_{h_z}^{zz} &amp;\Lambda_{1}^{zy} &amp; \cdots &amp; \Lambda_{h_y}^{zy}\nonumber \\[6pt] \Lambda_{2}^{zz} &amp; \cdots &amp; \Lambda_{h_z+1}^{zz} &amp;\Lambda_{2}^{zy} &amp; \cdots &amp; \Lambda_{h_y+1}^{zy}\nonumber \\[6pt] \vdots &amp; \vdots &amp; \vdots &amp; \vdots &amp; \vdots &amp; \vdots \nonumber\\[6pt] \Lambda_{h_z}^{zz} &amp; \cdots &amp; \Lambda_{2h_z-1}^{zz} &amp;\Lambda_{h_z}^{zy} &amp; \cdots &amp; \Lambda_{h_z+h_y-1}^{zy} \\[6pt] \hline \Lambda_{1}^{yz} &amp; \cdots &amp; \Lambda_{h_z}^{yz} &amp;\Lambda_{1}^{yy} &amp; \cdots &amp; \Lambda_{h_y}^{yy} \nonumber\\[6pt] \Lambda_{2}^{yz} &amp; \cdots &amp; \Lambda_{h_z+1}^{yz} &amp;\Lambda_{2}^{yy} &amp; \cdots &amp; \Lambda_{h_y+1}^{yy} \\[6pt] \vdots &amp; \vdots &amp; \vdots &amp; \vdots &amp; \vdots &amp; \vdots \nonumber\\ \Lambda_{h_y}^{yz} &amp; \cdots &amp; \Lambda_{h_y+h_z-1}^{yz} &amp;\Lambda_{h_y}^{yy} &amp; \cdots &amp; \Lambda_{2h_y-1}^{yy} \\[6pt] \end{array} \right). \end{align*}

We can then write **H**_*w*_ in terms of model parameters $\mathbf{A}, \mathbf{C}_{y}, \mathbf{C}_{z},\mathbf{G}_{y}, \mathbf{G}_{z}$, where $\mathbf{G}_{y} = \mathrm{Cov}[\mathbf{x}_{t+1},\mathbf{y}_{t}]$ and $\mathbf{G}_{z} = \mathrm{Cov}[\mathbf{x}_{t+1},\mathbf{z}_{t}]$. It can be shown (see appendix D) that for positive integer *k*’s we have $\Lambda_{k}^{yy} = \mathbf{C}_{y}\mathbf{A}^{k-1}\mathbf{G}_{y}$, $\Lambda_{k}^{zz} = \mathbf{C}_{z}\mathbf{A}^{k-1}\mathbf{G}_{z}$, $\Lambda_{k}^{yz} = \mathbf{C}_{y}\mathbf{A}^{k-1}\mathbf{G}_{z}$ and $\Lambda_{k}^{zy} = \mathbf{C}_{z}\mathbf{A}^{k-1}\mathbf{G}_{y}$. Replacing these values in equation (19), gives:

\begin{align*} \mathbf{H}_{w} = \left(\begin{array}{c|c} \mathbf{H}_{w}^{zz}&amp; \mathbf{H}_{w}^{zy}\\ \hline \mathbf{H}_{w}^{yz}&amp; \mathbf{H}_{w}^{yy} \end{array}\right) \end{align*}

with

\begin{equation*} \mathbf{H}_{w}^{zz} = \left( \begin{array}{cccc} \mathbf{C}_{z}\mathbf{G}_{z} &amp; \mathbf{C}_{z}\mathbf{A}\mathbf{G}_{z} &amp; \cdots &amp; \mathbf{C}_{z}\mathbf{A}^{h_z-1}\mathbf{G}_{z} \\ \mathbf{C}_{z}\mathbf{A}\mathbf{G}_{z} &amp; \mathbf{C}_{z}\mathbf{A}^{2}\mathbf{G}_{z} &amp; \cdots &amp; \mathbf{C}_{z}\mathbf{A}^{h_z}\mathbf{G}_{z}\\ \vdots &amp; \vdots &amp; \vdots &amp; \vdots\\ \mathbf{C}_{z}\mathbf{A}^{h_z-1}\mathbf{G}_{z} &amp; \mathbf{C}_{z}\mathbf{A}^{h_z}\mathbf{G}_{z} &amp; \cdots &amp; \mathbf{C}_{z}\mathbf{A}^{2h_z-2}\mathbf{G}_{z} \end{array} \right), \end{equation*}

\begin{equation*} \mathbf{H}_{w}^{zy} = \left( \begin{array}{cccc} \mathbf{C}_{z}\mathbf{G}_{y} &amp; \mathbf{C}_{z}\mathbf{A}\mathbf{G}_{y}&amp; \cdots &amp; \mathbf{C}_{z}\mathbf{A}^{h_y-1}\mathbf{G}_{y}\\ \mathbf{C}_{z}\mathbf{A}\mathbf{G}_{y} &amp; \mathbf{C}_{z}\mathbf{A}^{2}\mathbf{G}_{y} &amp; \cdots &amp; \mathbf{C}_{z}\mathbf{A}^{h_y}\mathbf{G}_{y}\\ \vdots &amp; \vdots &amp; \vdots &amp; \vdots\\ \mathbf{C}_{z}\mathbf{A}^{h_z-1}\mathbf{G}_{y} &amp; \mathbf{C}_{z}\mathbf{A}^{h_z}\mathbf{G}_{y} &amp; \cdots &amp; \mathbf{C}_{z}\mathbf{A}^{h_z+h_y-2}\mathbf{G}_{y} \end{array} \right), \end{equation*}

\begin{equation*} \mathbf{H}_{w}^{yz} = \left( \begin{array}{cccc} \mathbf{C}_{y}\mathbf{G}_{z} &amp; \mathbf{C}_{y}\mathbf{A}\mathbf{G}_{z} &amp; \cdots &amp; \mathbf{C}_{y}\mathbf{A}^{h_z-1}\mathbf{G}_{z} \\ \mathbf{C}_{y}\mathbf{A}\mathbf{G}_{z} &amp; \mathbf{C}_{y}\mathbf{A}^{2}\mathbf{G}_{z} &amp; \cdots &amp; \mathbf{C}_{y}\mathbf{A}^{h_z}\mathbf{G}_{z}\\ \vdots &amp; \vdots &amp; \vdots &amp; \vdots\\ \mathbf{C}_{y}\mathbf{A}^{h_y-1}\mathbf{G}_{z} &amp; \mathbf{C}_{y}\mathbf{A}^{h_y}\mathbf{G}_{z} &amp; \cdots &amp; \mathbf{C}_{y}\mathbf{A}^{h_y+h_z-2}\mathbf{G}_{z} \end{array} \right), \end{equation*}

\begin{equation*} \mathbf{H}_{w}^{yy} = \left( \begin{array}{cccc} \mathbf{C}_{y}\mathbf{G}_{y} &amp; \mathbf{C}_{y}\mathbf{A}\mathbf{G}_{y}&amp; \cdots &amp; \mathbf{C}_{y}\mathbf{A}^{h_y-1}\mathbf{G}_{y}\\ \mathbf{C}_{y}\mathbf{A}\mathbf{G}_{y} &amp; \mathbf{C}_{y}\mathbf{A}^{2}\mathbf{G}_{y} &amp; \cdots &amp; \mathbf{C}_{y}\mathbf{A}^{h_y}\mathbf{G}_{y}\\ \vdots &amp; \vdots &amp; \vdots &amp; \vdots\\ \mathbf{C}_{y}\mathbf{A}^{h_y-1}\mathbf{G}_{y} &amp; \mathbf{C}_{y}\mathbf{A}^{h_y}\mathbf{G}_{y} &amp; \cdots &amp; \mathbf{C}_{y}\mathbf{A}^{2h_y-2}\mathbf{G}_{y} \end{array} \right). \end{equation*}

It is easy to see from equation (20) that **H**_*w*_ can be decomposed as:

\begin{align*} {\mathbf{H}_{w} = \mathcal{O}_{w}\mathcal{C}_{w}} \end{align*}

with

\begin{align*} {\mathcal{O}_{w}\triangleq\left(\begin{array}{c} \mathcal{O}_{z}\\ \hline \mathcal{O}_{y} \end{array}\right)\triangleq\left(\begin{array}{c} \mathbf{C}_{z} \\ \mathbf{C}_{z}\mathbf{A} \\ \vdots \\ \mathbf{C}_{z}\mathbf{A}^{h_z-1} \\ \hline \mathbf{C}_{y} \\ \mathbf{C}_{y}\mathbf{A} \\ \vdots \\ \mathbf{C}_{y}\mathbf{A}^{h_y-1} \\ \end{array}\right)} \end{align*}

and

\begin{align*} {\mathcal{C}_{w} \triangleq\left(\begin{array}{c} \mathcal{C}_{z}^{^{\prime}}\\ \hline \mathcal{C}_{y}^{^{\prime}} \end{array} \right)^{^{\prime}}\triangleq \left(\begin{array}{c} \mathbf{G}_{z}^{^{\prime}} \\ \left(\mathbf{A}\mathbf{G}_{z}\right)^{^{\prime}} \\ \vdots \\ \left(\mathbf{A}^{h_z-1}\mathbf{G}_{z}\right)^{^{\prime}}\\ \hline \mathbf{G}_{y}^{^{\prime}} \\ \left(\mathbf{A}\mathbf{G}_{y}\right)^{^{\prime}} \\ \vdots \\ \left(\mathbf{A}^{h_y-1}\mathbf{G}_{y}\right)^{^{\prime}}\\ \end{array} \right)^{^{\prime}}.} \end{align*}

Here $\mathcal{O}_w$ ($\mathcal{C}_w$) is the multiscale extended observability (reachability) matrix, which is the concatenation of single-scale extended observability (reachability) matrices, $\mathcal{O}_z$ and $\mathcal{O}_y$ ($\mathcal{C}_z$ and $\mathcal{C}_y$).

This concludes how **H**_*w*_ is related to model parameters. Based on these relationships, we will next use the estimation of **H**_*w*_ obtained from real data (see section 2.2.3) to estimate all model parameters.

#### 2.2.5. Estimating model parameters from empirical estimates of **H**_*w*_, Λ_0_ and *µ* ^*z*^

Using the the following steps, we estimate the multiscale model parameters $\mathcal{N} = \{\mathbf{A},\mathbf{C}_{y},\mathbf{C}_{z},\mathbf{Q},\mathbf{R}_{y},\mathbf{d}_z\}$ from the estimated **H**_*w*_ and Λ_0_ and *µ* ^*z*^, which were estimated from the data via the transformation of moments technique (section 2.2.3):

(i) Find estimates of extended observability matrix $\mathcal{O}_{w}$ and extended reachability matrix $\mathcal{C}_{w}$ (equations (21)–(23)) by applying SVD on the estimated **H**_*w*_ and keeping only the largest *n_x_* singular values:
  \begin{align*} \mathbf{H}_{w} = \mathbf{U}\mathbf{K}\mathbf{V}^{\prime}\approx\mathbf{U}_1\mathbf{K}_1\mathbf{V}_1^{\prime}. \end{align*}
  Here $\mathbf{K}_1 \in \mathbb{R}^{n_x \times n_x}$ is a diagonal matrix containing the *n_x_* largest singular values, and $\mathbf{U}_1 \in \mathbb{R}^{(h_yn_y+h_zn_z) \times n_x}$ and $\mathbf{V}_1 \in \mathbb{R}^{(h_yn_y+h_zn_z) \times n_x}$ are the associated left and right singular vectors, respectively.
  We then have:
  \begin{align*} \mathcal{O}_{w} = \left( \begin{array}{c} \mathcal{O}_{z}\\ \mathcal{O}_{y} \end{array} \right) = \mathbf{U}_1\mathbf{K}_1^{\frac{1}{2}}, \end{align*}
  and
  \begin{align*} \mathcal{C}_{w} = \left( \begin{array}{cc} \mathcal{C}_{z}&amp; \mathcal{C}_{y} \end{array} \right) = \mathbf{K}_1^{\frac{1}{2}}\mathbf{V}_1^{^{\prime}}. \end{align*}
(ii) Extract estimates of **C**_*y*_ and **C**_*z*_ as the first *n_y_* and *n_z_* rows of estimates of $\mathcal{O}_y$ and $\mathcal{O}_z$ from step (i), respectively (see equation (22)):
  \begin{eqnarray*} \mathbf{C}_z = \mathcal{O}_z\left(1:n_z,:\right) \end{eqnarray*}
  \begin{eqnarray*} \mathbf{C}_y = \mathcal{O}_y\left(1:n_y,:\right) \end{eqnarray*}
  where : is used to indicate selecting all elements along a given row or column. Further $m:n$ indicates selection of elements ranging from the *m*th to the *n*th position along a row or column.
(iii) Estimate **A** by solving the following optimization problem:
  \begin{align*} \min_\mathbf{A} \|\underline{\mathcal{O}_{z}}\mathbf{A}-\overline{\mathcal{O}_{z}}\|_\mathrm{F}^{2}+ \|\underline{\mathcal{O}_{y}}\mathbf{A}-\overline{\mathcal{O}_{y}}\|_\mathrm{F}^{2}, \end{align*}
  where
  \begin{align*} \underline{\mathcal{O}_{y}} &amp; = \mathcal{O}_{y}\left(1:\left(h_y-1\right)n_y,:\right), \end{align*}
  \begin{align*} \overline{\mathcal{O}_{y}} &amp; = \mathcal{O}_{y}\left(n_y+1:h_yn_y,:\right), \end{align*}
  \begin{align*} \underline{\mathcal{O}_{z}} &amp; = \mathcal{O}_{z}\left(1:\left(h_z-1\right)n_z,:\right), \end{align*}
  \begin{align*} \overline{\mathcal{O}_{z}} &amp; = \mathcal{O}_{z}\left(n_z+1:h_zn_z,:\right), \end{align*}
  and $\|.\|_\mathrm{F}$ represents the Frobenius norm.
  The optimization problem in (29) combines information across modalities, i.e. multimodal discrete-continuous data, through the cost function which sums up squared error of finding **A** from $\mathcal{O}_z$ and from $\mathcal{O}_y$ (see equation (22)). The optimization problem in (29) can also be written as:
  \begin{eqnarray*} \min_\mathbf{A} \|\left( \begin{array}{c} \underline{\mathcal{O}_{z}}\\ \underline{\mathcal{O}_{y}} \end{array} \right) \mathbf{A}- \left( \begin{array}{c} \overline{\mathcal{O}_{z}}\\ \overline{\mathcal{O}_{y}} \end{array} \right) \|_\mathrm{F} \end{eqnarray*}
  which has the following analytical least square solution:
  \begin{align*} A = \left( \begin{array}{c} \underline{\mathcal{O}_{z}}\\ \underline{\mathcal{O}_{y}} \end{array} \right)^{\dagger} \left( \begin{array}{c} \overline{\mathcal{O}_{z}}\\ \overline{\mathcal{O}_{y}} \end{array} \right). \end{align*}
(iv) Extract **G**_*z*_ and **G**_*y*_ as the first *n_y_* and *n_z_* columns of estimates of $\mathcal{C}_{z}$ and $\mathcal{C}_{y}$ from step (i) (see equation (23)):
  \begin{align*} \mathbf{G}_{z} = \mathcal{C}_{z}\left(:,1:n_z\right), \end{align*}
  and
  \begin{align*} \mathbf{G}_{y} = \mathcal{C}_{y}\left(:,1:n_y\right). \end{align*}
(v) Estimate valid noise covariances **Q** and **R**_*y*_ by solving the following convex constrained optimization problem, which addresses the second challenge in developing multiscale SID (section 2.2.2):
  \begin{align*} \left\{ \begin{array}{@{}l} \min_{\Sigma_{x}} \|\mathbf{S}\left(\Sigma_{x}\right)\|_\mathrm{F}+\|\mathbf{R}_{z}\left(\Sigma_{x}\right)\|_\mathrm{F}+\|\mathbf{R}_{zy}\left(\Sigma_{x}\right)\|_\mathrm{F}\\[5pt] \mbox{such that}~\Sigma_{x},~\mathbf{Q}\left(\Sigma_{x}\right),~\mathbf{R}_{y}\left(\Sigma_{x}\right)~ \mbox{are PSD.} \end{array} \right. \end{align*}
  where
  \begin{align*} \left\{ \begin{aligned} \mathbf{Q}\left(\Sigma_{x}\right)&amp; = \Sigma_{x}-\mathbf{A}\Sigma_{x}\mathbf{A}^{^{\prime}},\\[3pt] \mathbf{R}\left(\Sigma_{x}\right)&amp;\triangleq\left(\begin{array}{cc}\mathbf{R}_{z}\left(\Sigma_{x}\right) &amp; \mathbf{R}_{zy}\left(\Sigma_{x}\right)\\ [3pt]\mathbf{R}_{zy}^{^{\prime}}\left(\Sigma_{x}\right) &amp; \mathbf{R}_{y}\left(\Sigma_{x}\right) \end{array}\right)\\[3pt] ~&amp; = \Lambda_{0}-\left(\begin{array}{c} \mathbf{C}_{z}\\ \mathbf{C}_{y}\end{array}\right) \Sigma_{x} \left(\begin{array}{c}\mathbf{C}_{z}\\ \mathbf{C}_{y}\end{array}\right)^{^{\prime}},\\[3pt] \mathbf{S}\left(\Sigma_{x}\right)&amp; = \left(\begin{array}{cc} \mathbf{G}_{z}&amp; \mathbf{G}_{y}\end{array}\right)-\mathbf{A}\Sigma_{x} \left(\begin{array}{c}\mathbf{C}_{z}\\ \mathbf{C}_{y}\end{array}\right)^{^{\prime}} \end{aligned} \right. \end{align*}
  with $\Sigma_{x}\triangleq \mathrm{Cov}(\mathbf{x}_{t})$, $\Lambda_{0} = \mathrm{Cov}[(\begin{array}{c}\mathbf{z}_{t}\\ \mathbf{y}_{t}\end{array})]$.
  Note that the estimates for all the model parameters that are required to solve this optimization problem—i.e. **A**, **C**_*y*_, **C**_*z*_, **G**_*y*_, **G**_*z*_, Λ_0_—are available from previous steps. Also, equations in (39), can be derived from the model in equation (2) (see appendix E). For more details and a description of how this step (along with step (vi)) addresses the second challenge in developing multiscale SID see section 2.2.6.
(vi) Update estimates of **G**_*y*_, **G**_*z*_, Λ_0_: given the solution for $\Sigma_{x}$ obtained from solving the constrained optimization in the previous step, we set **R**_*z*_, $\mathbf{R}_{zy}$ and **S** to exactly 0 in equation set (39), and get updated estimates for Λ_0_, **G**_*y*_ and **G**_*z*_.
(vii) Read **d**_*z*_ from the estimated first moment of future–past log firing rate vector, i.e. $\mu^{z} \triangleq \mathbb{E}[\mathbf{z}_t^\mathrm{fp}]$ in section 2.2.3:
  \begin{align*} \mathbf{d}_z = \mu^{z}\left(1:n_z\right). \end{align*}

This concludes the learning of all multiscale model parameters $\mathcal{N} = \{\mathbf{A},\mathbf{C}_{y},\mathbf{C}_{z},\mathbf{Q},\mathbf{R}_{y},\mathbf{d}_z\}$. All steps of multiscale SID algorithm are summarized in algorithm 2.

Finally, we note that $\mathcal{P} = \{\mathbf{A},\mathbf{C}_{z},\mathbf{C}_{y},\mathbf{G}_{z}, \mathbf{G}_{y},\Lambda_0,$ $\mathbf{d}_z,\Sigma_x\}$ is also an alternative full specification of the multiscale model, which is equivalent to the specification with $\mathcal{N} = \{\mathbf{A},\mathbf{C}_{y},\mathbf{C}_{z},\mathbf{Q},\mathbf{R}_{y},\mathbf{d}_z\}$. This is due to the one to one relation between these two sets according to equations in (39) [23]. The model specification with $\mathcal{N}$ is useful when using the MSF for neural or behavior prediction (sections 2.3.4 and 2.4.3) [43], while $\mathcal{P}$ is more useful for model parameter evaluation (section 2.3.2).

#### 2.2.6. Ensuring validity of noise statistics

In steps (v)–(vi) of the parameter estimation procedure described in section 2.2.5, we addressed the key challenge of ensuring valid noise statistics in developing multiscale SID (section 2.2.2) by devising a convex constrained optimization. In this section, we provide more details and context for this novel approach.

The optimization problem in equation (38) aims to find noise statistics that satisfy the following conditions that are required in the multiscale model (equation (2)):

(i) State and continuous observation noise covariances **Q** and **R**_*y*_ must be valid covariance matrices, and thus need to be PSD.
(ii) State and continuous observation noises are assumed to be uncorrelated, i.e. $\mathbf{S} = 0$.
(iii) Log-firing rate is assumed not to have additive Gaussian noise, i.e. $\mathbf{R}_z = 0$ [43, 49] with the stochasticity of spiking data reflected in its Poisson distribution.

While the first condition of valid noise statistics is necessary for a valid model, the last two conditions are incorporated in the multiscale model (equation (2)) to be consistent with prior work on Poisson and multiscale modeling that required these conditions, often as an assumption to derive their inference method [6, 43, 49, 61, 63, 64, 72, 76, 87, 88]. Indeed, an MSF for a model without these conditions/assumptions is currently lacking in the literature. While developing new filters that can eliminate these assumptions is not the focus of our work, if such a filter is developed in the future, our framework can in principle be applied to learn the parameters of the associated model. This is because the constrained optimization that we formulate is general and flexible, and can remove or add various constraints and terms in the cost function. It is important to emphasize that if we were to abandon these assumptions during the learning of the multiscale model, then we could not use the model for predicting neural activity and behavior as a multiscale filter without such assumptions is currently lacking in the literature. That is why we impose these assumptions in our formulation. We show that despite these assumptions, our model can fuse information across the spike-field modalities for better decoding compared with spikes or field potentials alone (figure 8). Further, it can perform well on real NHP data while being significantly more computationally efficient than multiscale EM in training (figures 7).

To encourage solutions that satisfy the above conditions as closely as possible, in the optimization problem of equation (38), we minimize the sum of norms of $\mathbf{S}(\Sigma_x)$, $\mathbf{R}_{z}(\Sigma_x)$ and $\mathbf{R}_{zy}(\Sigma_x)$, subject to the required condition that $\Sigma_x$, $\mathbf{Q}(\Sigma_x)$ and $\mathbf{R}_{y}(\Sigma_x)$ are PSD. We find the numerical solution of $\Sigma_{x}$ in the convex constrained optimization problem in (38) [83, 84] using CVX [82], a MATLAB software that uses a disciplined convex programming approach [89]. We then get estimates of **Q**, **R**_*y*_ according to equation set (39). Alternatively, one could form and solve a similar constrained optimization problem using generic numerical optimization tools rather than using semi-definite programming via CVX [82]; the former may be less accurate but useful if equation (39) gives an infeasible convex optimization problem. This might happen if **H**_*w*_ and subsequently other parameters before noise statistics estimation are poorly estimated due to short length of data or low signal-to-noise-ratio (SNR).

The fundamental reason why we have the flexibility to find alternative sets of noise statistics that satisfy conditions such as the above three is that the states in the multiscale model (equation (2)) are latent and thus there are infinitely many equivalent solutions with different latent states for describing the same observed multimodal data **y**_*t*_ and **N**_*t*_. These include, but are not limited to (see Faurre’s theorem in [70, 71]), all equivalent models obtained by left-multiplying the latent state with an arbitrary invertible matrix, also known as similarity transformations. These equivalent alternative models have different state covariance matrices $\Sigma_{x}$. The optimization problem in equation (38) aims to find one of these equivalent models that satisfies the required conditions as much as possible.

### 2.3. Validation of multiscale SID using simulations

#### 2.3.1. Simulating multimodal neural data

To validate our multiscale SID in numerical simulation, we randomly generate sets of multiscale model parameters $\mathcal{N} = \{\mathbf{A},\mathbf{C}_{y},\mathbf{C}_{z},\mathbf{Q},\mathbf{R}_{y},\mathbf{d}_z\}$ in equation (1) and then generate multimodal spike-field activity from these models. In random generation of the model parameters, we also set criteria for desired SNR of field features, bias and maximum firing rates of spikes, contribution of dynamical modes in each modality and range of frequency and magnitudes from which dynamical modes are randomly drawn, all of which will be explained later. Note that each dynamical mode corresponds to a pair of complex conjugate eigenvalues or a real eigenvalue of the state transition matrix **A** (figure 1(d)).

Prior work suggests that there exist both shared and distinct dynamical modes in spiking and field potential activity [49]. Motivated by this and also to cover a general scenario, we simulate both shared and distinct modes. Distinct spike (field) modes are those that are only present in spiking (field) activity and shared modes are those that are present in both modalities of neural activity (both spiking and field potential activity). To quantify the presence of a mode in the dynamics of a modality, we define the contribution of the dynamical mode *i* to the dynamics of a modality $\mathrm{cntrb}_{\mathrm{modality},i}$ as the total variance of the activity in that modality that is generated from that mode (across all neural dimensions from that modality, either log firing rates **z**_*t*_ or field potential activity **y**_*t*_), i.e.:

\begin{align*} \left\{ \begin{aligned} \mathrm{cntrb}_{z,i}&amp; = \mathrm{trace}\left(\overline{\mathbf{C}}_{z,i}\overline{\Sigma}_{x,i}\overline{\mathbf{C}}_{z,i}^{^{\prime}}\right),\\[4pt] \mathrm{cntrb}_{y,i}&amp; = \mathrm{trace}\left(\overline{\mathbf{C}}_{y,i}\overline{\Sigma}_{x,i}\overline{\mathbf{C}}_{y,i}^{^{\prime}}\right) \end{aligned}\right. \end{align*}

where $\overline{\Sigma}_{x,i}$ is the covariance of states corresponding to mode *i*, which is a submatrix of state covariance $\Sigma_{x}$ (equation (54), appendix F). Further, $\overline{\mathbf{C}}_{z,i}$ ($\overline{\mathbf{C}}_{y,i}$) is a submatrix of **C**_*z*_ (**C**_*y*_) with columns corresponding to mode *i*. Note that, without loss of generality, **A** in simulation is generated in the block diagonal format (see item (ii) below). Finally, we denote the contribution of mode *i* to the dynamics of a modality normalized by the sum of contribution of all the modes as $\mathrm{nCntrb}_{\mathrm{modality},i}$.

To generate the multiscale model parameters $\mathcal{N} = \{\mathbf{A},\mathbf{C}_{y},\mathbf{C}_{z},\mathbf{Q},\mathbf{R}_{y},\mathbf{d}_z\}$ in equation (1), we proceed as follows:

(i) Generate $\mathbf{Q}\in\mathbb{R}^{n_x\times n_x}$: the diagonal entries of the diagonal **Q** matrix are absolute values of samples drawn from the standard normal distribution.
(ii) Generate $\mathbf{A}\in\mathbb{R}^{n_x\times n_x}$: we generate dynamical modes as $r\mathrm{e}^{j\theta}$, with randomly choosing *r* between $[0.950,0.999]$, and *θ* between $[0,0.0316]$, consistent with ranges observed in prior work modeling the motor cortical activity of NHPs [49]. *r* and *θ* determine the damping and oscillatory behavior of the dynamics. Complex modes appear as complex conjugate eigenvalues of **A**. We construct **A** in a block diagonal format where the block corresponding to mode *i* is $\left(\begin{array}{cc} r\text{cos}\theta&amp; -r\text{sin}\theta\\ r\text{sin}\theta&amp; r\text{cos}\theta \end{array}\right)$. Note that the block diagonal construction of **A** does not pose any loss of generality since all models can be converted to this form (known as the canonical form) via a similarity transformation [90].
(iii) Generate $\mathbf{C_y}\in\mathbb{R}^{n_y\times n_x}$ and $\mathbf{C_z}\in\mathbb{R}^{n_z\times n_x}$: randomly generate entries of these matrices according to a uniform distribution. Then, scale columns and rows of these matrices to enforce the desired maximum firing rate for each neuron and the required contributions of each mode, $\mathrm{cntrb}_{\mathrm{modality},i}$ based on its type (see appendix G for details). The maximum and bias of the firing rates are randomly and uniformly picked in the ranges $[40 \,\mathrm{Hz},50 \,\mathrm{Hz}]$ and $[5\, \mathrm{Hz},10\, \mathrm{Hz}]$, respectively.
(iv) Generate $\mathbf{R}_y\in\mathbb{R}^{n_y\times n_y}$: having set **C**_*y*_ and $\Sigma_x$ (appendix F), we set diagonal entries of the diagonal **R**_*y*_ to achieve the desired SNRs for **y**_*t*_. The SNR vector of field potential features is defined as $\mathrm{diag}(\mathbf{C}_y\Sigma_x\mathbf{C}_{y}^{\prime})./\mathrm{diag}(\mathbf{R}_y)$ and the entries are randomly picked in the range $[0.8, 1.2]$. Here, $\mathrm{diag}(.)$ denotes the operation of transforming the diagonal entries of a matrix into a vector, and ‘./’ is an element-wise division operator.

Given the multiscale model parameters $\mathcal{N} = \{\mathbf{A},\mathbf{C}_{y},\mathbf{C}_{z},\mathbf{Q},\mathbf{R}_{y},\mathbf{d}_z\}$ from equation (1), we can generate the multimodal spiking activity **N**_*t*_ for $t\in\{1,2,3{\ldots},T\}$ and field potential activity **y**_*t*_ for $t\in\{1,M+1,2M+1,{\ldots},T\}$ as follows. We set $\mathbf{x_0} = 0$ and generate **q**_*t*_ and $\mathbf{r}_{y,t}$ from zero-mean Gaussian distributions with covariance of **Q** and $\mathbf{R_y}$, respectively. We then generate **x**_*t*_, **y**_*t*_ and **z**_*t*_ by iterating through equation (1) for *t* = 1 to *t* = *T*. We set *M* = 5 here and discard field potentials at the intermittent time steps as missing observations. **N**_*t*_ is then generated from Poisson distributions with rates equal to the elements of the vector $\mathrm{exp}(\mathbf{z}_t)$. Finally, it is worth noting that the neural observation dimensions, i.e. dimensions of **N**_*t*_ or **y**_*t*_, can be provided in any order to our multiscale SID (e.g. adjacent data dimensions do not need to correspond to adjacent electrodes) and a model will be learned that appropriately accounts for that ordering. Consistent with this, we did not need to impose any explicit spatial structure assumption in our simulation and could keep them general.

#### 2.3.2. Quantifying parameter identification error

There are infinitely many equivalent latent state models for the multiscale model in equation (1), for example any invertible linear mapping of the latent state is a similarity transform that gives an equivalent model [23, 70, 71]. To take this into account when evaluating the learned models in simulations, we first find the similarity transform that makes the learned model as close as possible to the true model in simulations in terms of the basis of the latent state as also done in prior work [23] (see appendix H for details). We then compare the model parameters for the transformed learned model with the true model parameters. Note that this procedure does *not* change the learned model, rather only gives a different equivalent formulation for it so that it can be compared with the true model [23].

Given the true and the learned model parameters, we quantify the parameter identification error of a matrix/vector parameter *ψ* as:

\begin{align*} \mathrm{normalized\ error}\ \left(\psi\right) = \frac{\|\psi^\mathrm{id}-\psi^\mathrm{true}\|_\mathrm{F}}{\|\psi^\mathrm{true}\|_\mathrm{F}}, \end{align*}

where $\|.\|_\mathrm{F}$ represents Frobenius norm and $\psi^\mathrm{true}$ and $\psi^\mathrm{id}$ refer to the true and identified parameter values, respectively. We evaluate our multiscale SID algorithm by computing the normalized error for each $\psi\in\mathcal{P}$ except for $\Sigma_x$. This is because according to Faurre’s theorem [23, 70, 71], all the model parameters in $\mathcal{P}$ other than $\Sigma_x$ are uniquely determined from **y**_*t*_ and **N**_*t*_ up to within a similarity transform. $\Sigma_x$ on the other hand is a redundant description (or internal description, see [71]) for the observations and may have infinitely many solutions even beyond similarity transforms for the same observations [23, 71], and thus even a perfect learning method may not need to learn the same $\Sigma_x$ (within a similarity transform) as the true model [23].

We also compute the normalized error for the vector of eigenvalues of **A**; i.e. $\psi = \mathrm{eig}(\mathbf{A})$, and denote it as normalized mode error (see appendix I for more details). Note that the vector of eigenvalues of **A** does not change after similarity transformation up to within permutations. Furthermore, in addition to computing the normalized mode error for all the modes at once, we can compute it separately for each mode type—distinct spike modes, distinct field modes and shared modes (section 2.3.1)—to investigate how multiscale SID identifies each and the collective dynamics of both modalities.

For this analysis, we simulate 50 multiscale dynamical models according to section 2.3.1 with dimension of spiking activity *n_z_* randomly picked in the interval $[10,30]$, and set dimension of field potential activity $n_y = 4\times n_z$. We also set number of dynamical modes to four, with two shared modes, one distinct spike mode and one distinct field mode. To evaluate the effect of training sample size on these errors, we generate multimodal spiking and field potential activity with different sample sizes $T\in[1,2,10,50,100,1000]\times10^3$ from each model.

#### Quantifying the norm of cost function terms **R**_*z*_, $\mathbf{R}_{zy}$, and **S**

2.3.3.

The multiscale model (equation (1)) and its inference structure [43] require **R**_*z*_, $\mathbf{R}_{zy}$, and **S** to be zero and the optimization problem in equation (38) aims to satisfy these conditions. To evaluate how close to zero the identified **R**_*z*_, $\mathbf{R}_{zy}$, and **S** are, we normalize their Frobenius norm with the total covariance of the relevant terms, which are given by $\Lambda_{0}^{zz}\triangleq\mathrm{Cov}[\mathbf{z}_{t}]$, $\Lambda_{0}^{yy}\triangleq\mathrm{Cov}[\mathbf{y}_{t}]$, $\Lambda_{0}^{zy}\triangleq\mathrm{Cov}[\mathbf{z}_{t},\mathbf{y}_{t}]$, and $\left(\begin{array}{cc}\mathbf{G}_{z}&amp; \mathbf{G}_{y}\end{array}\right)\triangleq\mathrm{Cov}[\mathbf{x}_{t+1},\left(\begin{array}{c}\mathbf{z}_{t}\\ \mathbf{y}_{t}\end{array}\right)]$, respectively. Using the model equations, these can be computed as $\Lambda_{0}^{zz} = \mathbf{C}_{z}\Sigma_x\mathbf{C}_{z}^{^{\prime}}+ \mathbf{R}_{z}$, $\Lambda_{0}^{zy} = \mathbf{C}_{z}\Sigma_x\mathbf{C}_{y}^{^{\prime}}+ \mathbf{R}_{zy}$, and $\left(\begin{array}{cc}\mathbf{G}_{z}&amp; \mathbf{G}_{y}\end{array}\right) = \mathbf{A}\Sigma_{x} \left(\begin{array}{c}\mathbf{C}_{z}\\ \mathbf{C}_{y}\end{array}\right)^{^{\prime}}+\mathbf{S}$. We then evaluate the closeness of these normalized values to zero. Finally, as a control, we also normalize the norm of **R**_*y*_ with that of $\Lambda_{0}^{yy} = \mathbf{C}_{y}\Sigma_x\mathbf{C}_{y}^{^{\prime}}+ \mathbf{R}_{y}$ to confirm that this normalized norm does not converge to zero as **R**_*y*_ is not in the cost function.

#### 2.3.4. One-step-ahead prediction of spiking and field potential activity

To obtain the one-step-ahead prediction of spiking and field potential activity, we need to obtain the one-step-ahead prediction of latent states as a first step. To do so, we use the identified model parameters to construct the optimal filters to obtain the one-step-ahead prediction of latent states (figure 1(c)). The optimal filters for the single-modal spiking activity, the single-modal field potential activity and the multimodal spiking and field potential activity are the point process filter (PPF), the Kalman filter (KF) and the MSF, respectively. MSF is derived in our prior work and in special cases when only one of the two signals is observed, it reduces to either PPF or KF [43]. MSF can also simultaneously admit modalities that have different sampling rates by treating the intermediate samples of the slower modality as missing, thus not requiring interpolation [43]. We denote the one-step-ahead prediction of latent states at time step *t* as $\mathbf{x}_{t|t-1}$, where $\mathbf{O}_{t|t-1}$ denotes an estimation of **O**_*t*_ based on all neural observations up to time step *t* − 1.

Given the one-step-ahead prediction of latent states $\mathbf{x}_{t|t-1}$, the one-step-ahead prediction of field potentials is $\mathbf{y}_{t|t-1} = \mathbf{C}_y\mathbf{x}_{t|t-1}$. We use Pearson’s correlation coefficient (CC) between the one-step-ahead predicted field potential activity $\mathbf{y}_{t|t-1}$ and the true field potential activity **y**_*t*_, averaged over dimensions of field potential activity *n_y_*, as the accuracy measure for the one-step-ahead prediction of field potential activity [49].

We also obtain the one-step-ahead prediction of log firing rate as $\mathbf{z}_{t|t-1} = \mathbf{C}_z\mathbf{x}_{t|t-1}+\mathbf{d}_z$. We then compute the one-step-ahead predicted probability of having at least one spiking event in each time step (bin) based on the Poisson distribution for spiking activity. We then use different thresholds on this probability to predict whether a time step (bin) contained at least one spiking event (if the predicted probability is above the threshold). We compute the true positive rates and false positive rates by comparing to the actual spiking events to construct the receiver operating curve (ROC). We then find the area under the curve of ROC (AUC) as an accuracy measure for prediction of spiking activity for each neuron (spiking dimension). We compute a metric called prediction power $\mathrm{(PP)} = 2\times\mathrm{AUC}-1$ such that 0 is chance level and 1 is perfect prediction [6, 47, 49]. We report the PP, averaged over spiking dimensions *n_z_*, as the accuracy measure for the one-step-ahead prediction of spiking activity.

In these set of simulations we compute one-step-ahead prediction of spiking and field potential activity on a test set with 10^4^ samples, using the identified model parameters from the training set.

#### 2.3.5. Comparison of multiscale SID and multiscale EM in training time and accuracy

We compare the multiscale SID with the multiscale EM, which is the current method for learning the multiscale dynamical model [48, 49]. We perform comparisons in terms of training time and accuracy in identifying the dynamics and in one-step-ahead prediction of spiking and field potential activity. We continue the EM iterations until the following convergence criterion is met or until the number of iterations has reached a predefined maximum allowable number of iterations, which is set to 175 iterations here. We set the convergence criterion of the multiscale EM by putting a threshold on the relative change of a performance measure *m* in two consecutive steps, i.e.:

\begin{align*} |\frac{m^{\left(i\right)}-m^{\left(i+1\right)}}{m^{\left(i\right)}}|&lt;\mathrm{threshold} \end{align*}

where $m^{(i)}$ represents the performance measure *m* at iteration *i*. In this simulation analysis we take the normalized mode error (see section 2.3.2) as the performance measure *m* and we set the threshold to 10^−4^.

To compare the training time of multiscale SID vs. multiscale EM, we report the time it takes to learn the model parameters by each of the algorithms on the same computer. Further, to compare the performance of these algorithms in terms of identification of dynamics, we report the normalized mode error (section 2.3.2). Finally, we compare the accuracy in one-step-ahead prediction of spiking and field potential activities, which are quantified by PP and CC, respectively (section 2.3.4). These variables are reported for different training sample sizes for the same 50 simulated multiscale models as in section 2.3.2.

#### 2.3.6. Comparison of multiscale SID and single-scale SID in identification of dynamics

To demonstrate the potential benefit of the multiscale modeling over single-scale modeling for identification of dynamics, we perform an analysis where we combine neural signal of one modality with the other modality in simulated data.

We first simulate 50 multiscale dynamical models (equation (1)) with random parameters according to section 2.3.1, with $n_z = 14$ spiking signals, $n_y = 14$ field potential signals, and a combination of the same number of shared and distinct modes as previous sections, i.e. two shared modes, one distinct spike mode and one distinct field mode. We generate multimodal spiking and field potential activity with $T = 10^5$ samples from each model. Then, we construct and model sub-networks of the simulated multimodal network activity by gradually including more signals from one modality (either spiking or field potential activity, $n_\mathrm{add}\in\{2,4,{\ldots},14\}$), while keeping a fixed number of neural signals from the other modality, denoted as baseline neural signals. We set the number of baseline neural signals $n_\mathrm{baseline} = 4$, 6 or 14. We study how the learning error for the dynamics, quantified by normalized mode error (see section 2.3.2), changes as we increasingly include more signals from one modality of neural activity together with signals from the other modality (baseline signals). In addition, we study how the normalized mode error changes for each mode type—i.e. distinct spiking or field modes versus shared modes—separately (sections 2.3.1 and 2.3.2). With this we demonstrate how multiscale modeling helps to model the collective dynamics of both modalities, which includes both the modes that are only present in one of the two modalities and the shared modes that are present in both modalities. In this analysis, when both modalities are observed, we use our multiscale SID; when only baseline field potential or only baseline spiking activity is observed, we use the traditional single-scale SID algorithm for Gaussian observations [70] or the single-scale SID algorithm for Poisson observations [72], respectively.

Finally, we also compare the improvement gained by going from single-scale to multiscale modeling for cases with different number of baseline signals $n_\mathrm{baseline}$ to determine the baseline regimes in which multiscale modeling provides the greatest benefits. We quantify this improvement for each $n_\mathrm{baseline}$ baseline signals as the difference between the single-scale modeling error with $n_\mathrm{baseline}$ baseline signals and the minimum error among the models learned with combinations of $n_\mathrm{baseline}$ and $n_\mathrm{add}\in\{0,2,4,{\ldots},14\}$.

### 2.4. Validation of multiscale SID using NHP dataset

#### 2.4.1. Neural and behavioral recordings

We model the neural and behavioral data recorded from a male NHP (Monkey J), as it performed naturalistic 3D reach and grasp movements for a liquid reward (figure 7(a), see [23, 49, 78] for more details). All surgical and experimental procedures were in compliance with National Institute of Health Guide for Care and Use of Laboratory Animals and were approved by New York University Institutional Animal Care and Use Committee. A 137 electrode microdrive (Gray Matter Research, USA) was used to record spiking and LFP activity from left hemisphere motor cortical areas, covering parts of the primary motor cortex, the dorsal premotor cortex, the ventral premotor cortex, and the prefrontal cortex. Angle of multiple joints in the active arm (right) were inferred from the tracked position of retroreflective markers placed on the arm by using an NHP musculoskeletal model and inverse kinematics (SIMM, MusculoGraphics Inc. USA) [91]. We predict the angle of the following seven prominent joints in our analyses: shoulder elevation, elevation angle, shoulder rotation, elbow flexion, pro supination, wrist flexion, and wrist deviation [49].

#### 2.4.2. Neural data processing

To obtain the spiking activity (**N**_*t*_), spiking events were detected every time the band pass filtered raw neural signals (filtered within 0.3–6.6 kHz) crossed a threshold of 3.5 standard deviations below their mean [78], and were counted in 10 ms bins to get **N**_*t*_. Note that we do not sort recorded spikes from each electrode as customary in BMIs. We refer to this multiunit activity on each channel as one spiking signal. Future work could also study the relation of single-unit spiking activity to LFPs using the multiscale SID method. To obtain LFP features (**y**_*t*_), we first low pass filtered the raw neural signals with cut off frequency of 400 Hz and then down sampled it to 1 kHz. We then for each channel computed log-powers in seven frequency bands: theta (4–8 Hz), alpha (8–12 Hz), beta 1 (12–24 Hz), beta 2 (24–34 Hz), gamma 1 (34–55 Hz), gamma 2 (65–95 Hz), and gamma 3 (130–170 Hz) [43, 49, 59]. The log-power features were computed by first performing common average referencing, and then computing short-time Fourier transform for causal sliding windows of 300 ms every 50 ms. Thus, for our analyses, the time scale of LFP log-power features was 50 ms, and that of spike events was 10 ms [43, 49].

#### 2.4.3. Predicting behavior from the estimated latent states

To predict the behavior, i.e. joint angle trajectories, from the NHP neural data (section 2.4.1), we first use the learned models to estimate the low-dimensional latent states $\mathbf{x}_{t|t}$ from neural data, and then build a linear regression from these latent states to the behavior in the training set. To estimate the latent states $\mathbf{x}_{t|t}$, we use the learned model parameters to construct the associated optimal filters depending on the modality of neural activity observed in the model, i.e. KF, PPF or MSF [43] (figure 1(c)).

To build the regression model that predicts the behavior from latent states, we estimate the latent states within the training data and then compute the projection matrix **L**, which minimizes the mean squared error of predicting the behavior in the training data as $\mathbf{b}_{t} = \mathbf{L}\tilde{\mathbf{x}}_{t|t}$ [49]. Here, $\mathbf{b}_{t}\in\mathbb{R}^{n_b\times 1}$ is the behavior where $n_b = 7$ denotes its dimension, and $\tilde{\mathbf{x}}_{t|t} = [1,\mathbf{x}_{t|t}^{^{\prime}}]^{^{\prime}}$ is the estimated latent state vector concatenated with a constant to account for bias. The solution to this for this ordinary least squares linear regression problem is:

\begin{align*} \mathbf{L} = \mathbf{B}\mathbf{X}^{^{\prime}}\left(\mathbf{X}\mathbf{X}^{\prime}\right)^{-1} \end{align*}

where, $\mathbf{B} = [\mathbf{b}_1,{\ldots},\mathbf{b}_t,{\ldots},\mathbf{b}_T]\in\mathbb{R}^{n_b\times T}$, $\mathbf{X} = [\tilde{\mathbf{x}}_1,{\ldots},\tilde{\mathbf{x}}_t,{\ldots},\tilde{\mathbf{x}}_T]\in \mathbb{R}^{(n_x+1) \times T}$ and *T* is the size of training set. In the test set, we first estimate the latent states $\mathbf{x}_{t|t}$ using the appropriate filter (MSF, KF, or PPF), and then predict the behavior using the learned **L** projection matrix from the training set (figure 1(c)):

\begin{align*} \hat{\mathbf{b}}_t = \mathbf{L} \left[1,\mathbf{x}_{t|t}^{^{\prime}}\right]^{^{\prime}}. \end{align*}

We use Pearson’s CC between the predicted behavior and the true behavior, as the measure of behavior prediction accuracy. In our analysis, we report the mean of this CC over the seven joint angle trajectories.

#### 2.4.4. Five-fold cross-validation

In all the analyses for the NHP dataset, we use five-fold cross-validation. More precisely, we divide the data from each experimental session into five equal sized continuous sections, and in each fold, use four out of the five sections for training and use the remaining section for testing. We repeat this procedure five times so that each section has been used as the test data once. Further, we perform our analyses across seven experimental sessions from the subject [23, 49]. Finally, in each cross-validation fold, we *z*-score each dimension of the field potential activity based on its mean and variance within the training set [23]. This was done as a preemptive measure to ensure that learning methods do not discount any dimension of the field potential activity even if that dimension had a much smaller variance compared with other dimensions [23].

#### 2.4.5. Comparison of multiscale SID and multiscale EM in terms of training time and accuracy

We predict neural activity and behavior, i.e. the seven joint angle trajectories (section 2.4.1), from the NHP multimodal spiking and field potential activity using both multiscale SID and EM and compare these algorithms in terms of accuracy and training time. For this analysis, we construct the multimodal spiking and field potential activity that is to be modeled for each recording session by picking the top 15 spike channels and the top 15 LFP channels with highest behavior prediction accuracy when modeled as individual channels. The behavior prediction accuracy of the individual channels is computed and sorted using a basic non-latent KF decoder where the states are taken to be the behavior itself [43, 92]. We identify the multiscale model parameters by applying the multiscale SID (section 2.1) or the multiscale EM [48, 49] to the training data. For each learned model, we then estimate the latent states in the test data using the MSF associated with the learned model, and predict the neural activity (section 2.3.4) and the behavior (section 2.4.3) from the estimated latent states (figure 1(c)). We repeat this analysis for latent state dimensions $n_x\in\{2,4,{\ldots},24\}$. As with all other analyses, we set the horizon for multiscale SID as $h_y = h_z = 10$ (section 2.2.3). Finally, across different latent states, we compare the training time, i.e. the time it takes to learn the model parameters (similar to section 2.3.5), the one-step-ahead prediction accuracy of spiking and field potential activity, and the behavior prediction accuracy (defined in sections 2.3.4 and 2.4.3) between multiscale SID and multiscale EM algorithms. To determine the multiscale EM convergence, we set the measure *m* in equation (43) once to one-step-ahead prediction accuracy of field potential activity (CC) and once to that of spiking activity (PP) and take the larger *i* across the two as the convergence iteration. Similar to section 2.3.5, the multiscale EM is terminated when the convergence criterion is met or once we reach the predefined maximum allowable number of iterations, which we set to 150 iterations for this analysis.

#### 2.4.6. Comparison of multiscale SID and single-scale SID in predicting behavior

To investigate the potential benefit of multiscale modeling over single-scale modeling in the NHP dataset, we combine neural signals from different modalities, similar to what we do for simulated data (section 2.3.5). We then study the behavior prediction accuracy instead of identification of dynamical modes as the ground-truth of the latter is not known in real data.

In this analysis, we pick the top 30 spike channels and the top 30 LFP channels (210 LFP power features), which have the highest single channel behavior prediction accuracy when modeled individually, as the spiking and field potential activity to be modeled. We then randomly select $n_\mathrm{baseline}$ signals from one modality, denoted as baseline neural signals, and gradually combine additional randomly selected signals from the other modality of neural activity with them (in steps of $n_\mathrm{add}\in\{2,4,{\ldots},14\}$). We repeat this process of random selection of baseline and added neural signals 10 times for each of $n_\mathrm{baseline} = 2,\ 6 \ \mathrm{or} \ 14$. For each pair of baseline neural signals and added neural signals, we use the multiscale SID in combination with the MSF to estimate the latent states, predict the behavior and compute the behavior prediction accuracy, all within a five-fold cross-validation (figures 1(b) and (c), section 2.4.4). When evaluating models of baseline neural signals alone (not combined with the other modality of neural activity), we use the appropriate single-scale SID and single-scale filters to obtain the behavior prediction and compute their cross-validated accuracy (section 2.4.3). Given the computed behavior prediction accuracies for single-modal baseline neural signals, and for multimodal baseline and added neural signals together, we can study how multiscale modeling and filtering may help in behavior prediction compared to single-scale modeling and filtering. Additionally, we quantify the improvement of multimodal modeling compared to single-scale modeling for different baseline regimes similar to the simulation analysis in section 2.3.6.

For this analysis, we fit models for latent state dimensions $n_x\in\{2,5:5:20\}$. To select *n_x_* for a given fold and a given signal combination, we divide the training data for that fold into an inner training set consisting of $80\%$ of the training data and inner test set consisting of the remaining $20\%$ of the training data. We then learn the model parameters using the inner training set and use them to predict the behavior in the inner test set. We then choose the *n_x_* that results in the best behavior prediction accuracy on the inner test set.

### 2.5. Statistical analysis

All the statistical analyses for paired samples are done one-sided with Wilcoxon signed rank test. Significance is declared if the *P* < 0.05. In cases where multiple comparison are being made, we use the false discovery rate (FDR) control procedure from Benjamini–Hochberg [93] to correct for all comparisons and report the FDR-corrected *P* values.

## 3. Results

### 3.1. Simulation validations: multiscale SID performs accurately while being substantially more computationally efficient in training

We simulated multimodal discrete-continuous neural data from models with random parameters according to equation (1) (see section 2.3.1 for simulation details). We then applied the learning algorithms to the simulated data to learn the model parameters, identify dynamical modes, extract the latent states and predict neural activity (see sections 2.2.5, 2.3.4, figures 1(b)–(d)). Each dynamical mode corresponds to a pair of complex conjugate eigenvalues or a real eigenvalue of the state transition matrix **A**. To show that multiscale SID in figure 1(b) can successfully aggregate multimodal data, we compared it with single-scale SID algorithms for continuous field potentials alone [70] and for discrete spikes alone [72]. To show that multiscale SID achieves good accuracy while being substantially more computationally efficient in training time, we compared it with the existing multiscale EM algorithm for multimodal spike-field neural data [48, 49]. Performance measures are detailed in sections 2.3.2–2.3.4. Unless otherwise stated, all performance metrics, training times, and statistics are reported for the multiscale EM after convergence, and training time for EM refers to the time to convergence. Note that while we refer to the discrete-continuous data as spike-field for ease of exposition, these are general simulated multimodal data; thus, these simulations validate multiscale SID broadly for multimodal Poisson and Gaussian data observations.

#### 3.1.1. Multiscale SID accurately identifies the model parameters

**Figure 2.**
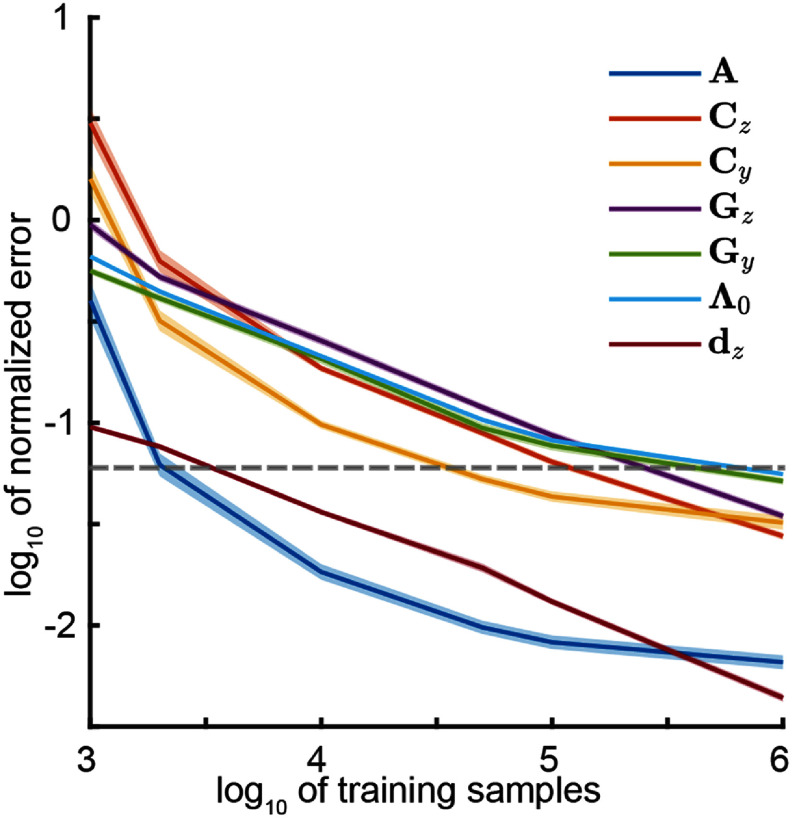
Multiscale SID accurately identifies the model parameters in simulations. Normalized identification error of all parameters in multiscale SID as a function of number of training samples across 50 randomly generated multiscale models. All parameter identification errors decrease as more training samples are used. Using 10^6^ samples, all normalized errors are less than $6\%$. The dashed horizontal line indicates $ 6\%$ normalized error. The normalized error is the Frobenius norm of the difference between the true and identified parameters, which is then normalized by the true parameter norm (equation (42)). Solid lines show the mean and shaded area represents s.e.m. The set $\{\mathbf{A},\mathbf{C}_{z},\mathbf{C}_{y},\mathbf{G}_{z}, \mathbf{G}_{y},\Lambda_0,\mathbf{d}_z\}$ fully characterize the multiscale model in equation (1), where $\Lambda_{0} = \mathrm{Cov}[(\begin{array}{cc}\mathbf{z}_{t}^{^{\prime}}&amp; \mathbf{y}_{t}^{^{\prime}}\end{array})^{^{\prime}}]$ is the covariance of concatenated log firing rates and field potential signals, and $\mathbf{G}_y = \mathrm{Cov}[\mathbf{x}_{t+1},\mathbf{y}_t]$ and $\mathbf{G}_z = \mathrm{Cov}[\mathbf{x}_{t+1},\mathbf{z}_t]$ are the cross-covariances of latent states with log firing rates and field potential signals, respectively (see sections 2.2.5 and 2.3.2).

We found that model parameters were identified with decreasing normalized error as the number of training samples increased (figure 2). The normalized error, defined in equation (42), is the Frobenius norm of the difference between true and identified parameters, which is then normalized by the true parameter norm. All model parameters could be identified accurately, with the normalized error reaching below $6\%$ when trained with $T = 10^6$ training samples (figure 2).

#### 3.1.2. The optimization problem satisfies the desired conditions

**Figure A1.**
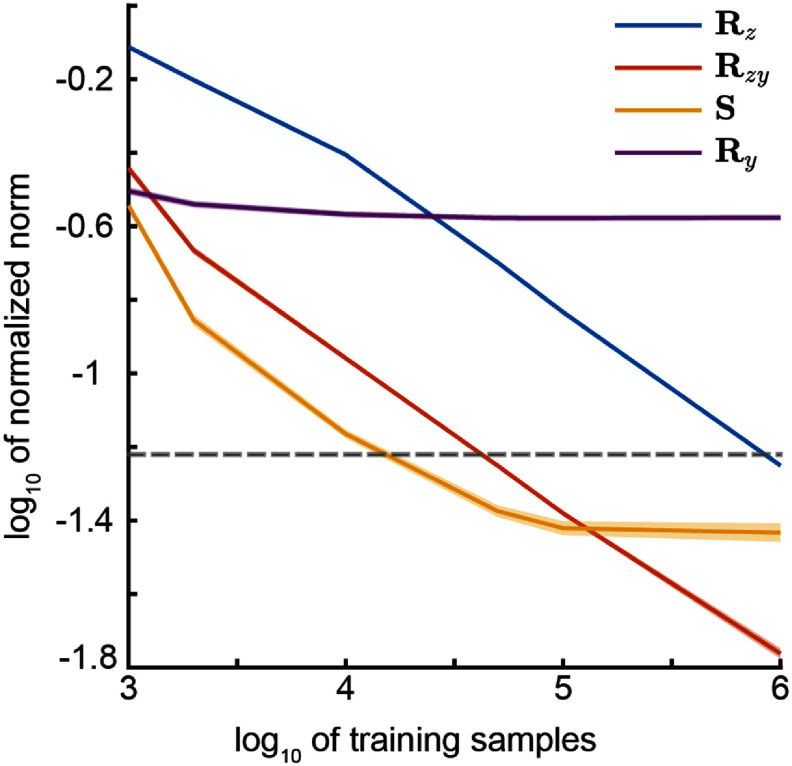
The optimization problem satisfies the desired conditions. Normalized norm of parameters **R**_*z*_, $\mathbf{R}_{zy}$ and **S** (defined in section 2.3.3) identified by multiscale SID as a function of number of training samples across 50 randomly generated multiscale models. All normalized norms decrease as more training samples are used. Using 10^6^ samples, all normalized norms are less than $6\%$. The dashed horizontal line indicates $ 6\%$ normalized norm. Since **R**_*y*_ is not in the cost function, its normalized norm is shown as a control to show that it is not driven to zero. Solid lines show the mean and shaded area represents s.e.m.

We also investigated the extent to which the terms **R**_*z*_, $\mathbf{R}_{zy}$, and **S** in the constrained optimization problem cost function (equation (38)), are driven to zero. We found that the normalized norm of **R**_*z*_, $\mathbf{R}_{zy}$ and **S** decreased as the number of training samples was increased, and all reached less than $6\%$ at $T = 10^6$ training samples (figure A1). Note that as the absolute norm of these matrices are not meaningful, we normalized them as described in section 2.3.3. Finally, as a control, we found that the normalized norm of **R**_*y*_, was not converging to zero with increasing training sample size, which is expected since **R**_*y*_ is not one of the terms in the cost function of the optimization problem (equation (38)). Note also that as we showed in figure 2, the normalized error of all model parameters decreases with increasing the training sample size, thus again confirming the success of the learning method including the constrained optimization.

#### 3.1.3. Multiscale SID outperforms multiscale EM in training time and in identification of dynamical modes, while reaching a similar neural prediction accuracy

**Figure 3.**
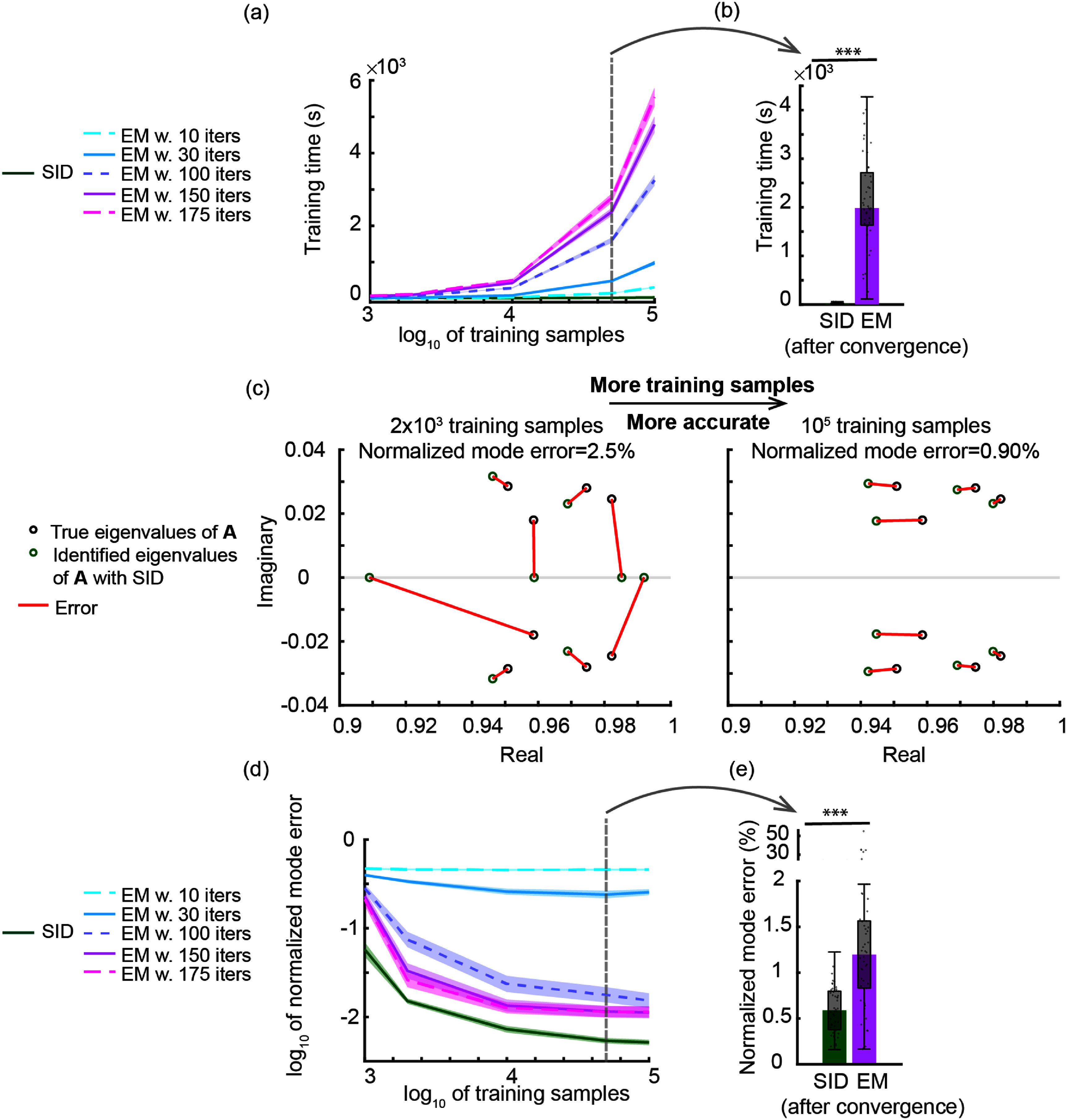
Multiscale SID outperforms multiscale EM in training time and identification of dynamical modes in simulations. (a) Training time (in seconds) of multiscale SID and multiscale EM with different number of iterations as a function of number of training samples. Multiscale SID has a much lower training time compared with multiscale EM. Also, the training time of multiscale EM monotonically increases with more iterations or training samples. Solid line represents the mean across 50 simulated models and shaded area represents s.e.m. (b) Training time of multiscale SID vs multiscale EM using $5\times10^4$ training samples (indicated by vertical dashed line on panel (a)). This training sample size has the same order of magnitude as the NHP dataset. Bars represent the median, box edges represent 25th and 75th percentiles, and whiskers show the minimum and maximum values (other than outliers). Outliers are the points that are more than 1.5 times the interquartile distance (the box length) away from the top and bottom of the box. The dots represent individual data points. Asterisks indicate significance of performance comparison between multiscale SID and multiscale EM. (Wilcoxon signed rank test, $N_\mathrm{s}$ = 50, *: *P* < 0.05, **: *P* < 0.005, ***: *P* < 0.0005). (c) For one simulated multiscale model, the true and SID identified eigenvalues of the state transition matrix **A** are shown in black and green circles for $2\times10^3$ (left) and $5\times10^4$ training samples (right). Red lines indicate the identified eigenvalue errors, that is mode errors. Each dynamical mode corresponds to a pair of complex conjugate or a real eigenvalue of the true state transition matrix **A** (figure 1(d)). Normalized mode error is computed by first dividing the sum of the squared length of the red error lines by the sum of the true eigenvalue squared magnitude, and then taking its square root (section 2.3.2). Normalized mode error decreases with increasing the training sample size. (d) and (e) Same as (b) and (c) but for normalized mode error. Multiscale SID has a significantly lower mode error compared with multiscale EM. The normalized mode error monotonically decreases with more iterations for multiscale EM and with more training samples for both multiscale EM and SID.

**Figure 4.**
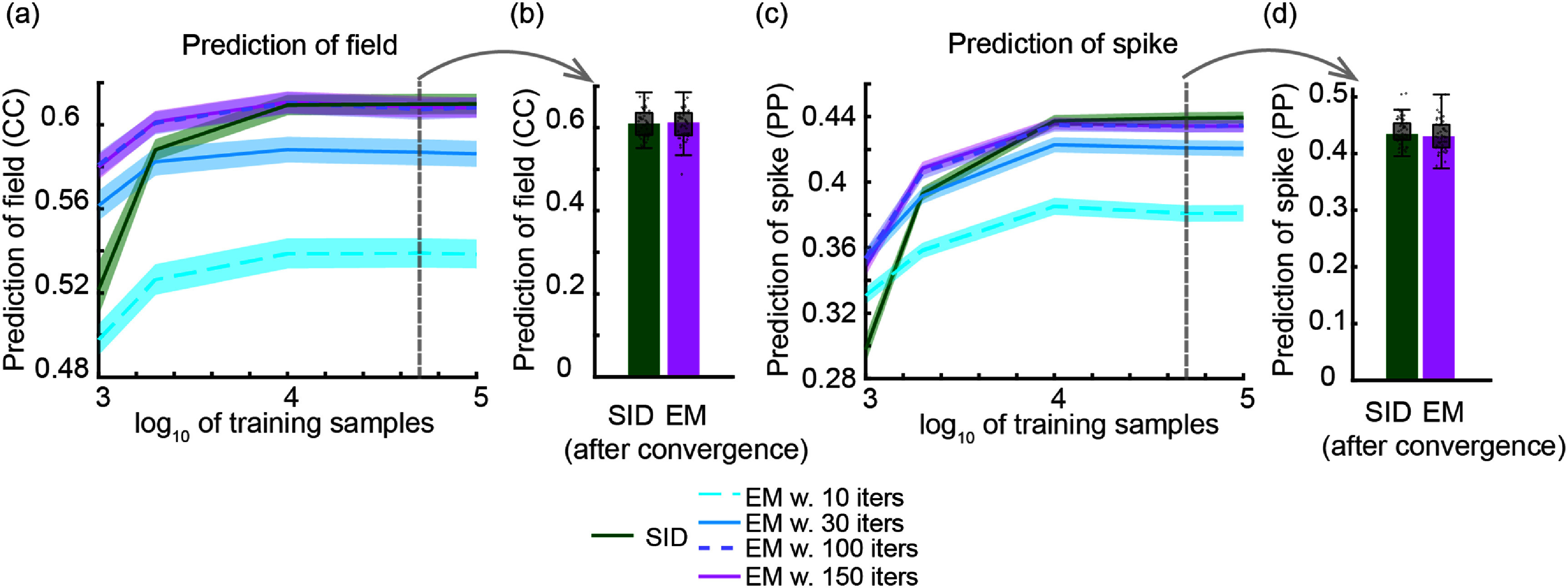
While allowing for significantly faster training time (figure 3), multiscale SID still has comparable performance to multiscale EM in predicting spiking and field potential data in simulations. (a) One-step-ahead prediction accuracy of field potential activity quantified by correlation coefficient (CC) between the one-step-ahead predicted and the true field potential activity for multiscale SID and multiscale EM (with different number of iterations) as a function of training samples. (b) One-step-ahead prediction performance of field potential activity for multiscale SID vs multiscale EM using $5\times10^{4}$ training samples, which is similar to the number of samples in the NHP datasets and indicated by the vertical dashed line on panel (a). (c) and (d) Same as (a) and (b) but for one-step-ahead prediction accuracy of spiking activity quantified by prediction power (PP, section 2.3.4). Figure convention is as in figure 3, and the performance is also reported for the same 50 simulated systems in that figure ($N_\mathrm{s} = 50$). Overall, multiscale SID performs similarly in neural prediction to multiscale EM in simulations when enough training samples—but still comparable to that of the real data—are available.

For the same 50 simulated systems as in section 3.1.1, we compared the training time of multiscale SID with that of multiscale EM in learning the model parameters as well as their performance in identifying dynamical modes and prediction of neural activity. We continued the multiscale EM iterations until convergence in dynamical mode identification error or up to 175 iterations, whichever happened earlier (see sections 3.1 and 2.3.5). We compute and compare the performances as a function of training sample size (figures 3 and 4). We also separately highlight the results for $T = 5\times10^4$ training samples, which is in the same order of magnitude as the session lengths of the NHP dataset used in this study ($2.79\times10^{4}\pm0.14\times10^{4}$ ($\mathrm{mean}\pm \mathrm{s.e.m.}$) samples).

*Training time*: First, the training time required for learning the model parameters in multiscale SID was orders of magnitude faster than that of multiscale EM (figures 3(a) and (b)). For example, for $T = 5\times10^4$ training samples, the training time for multiscale SID was $18.85 \pm 2.16$ s vs. $2127.80\pm 133.70$ s for multiscale EM, i.e. $186.00\pm 20.41$ times faster (figure 3(b), $P = 3.89\times10^{-10}$, $N_\mathrm{s} = 50$). Also, the medians for the two methods were 13.94 s vs 1983.50 s, respectively. Further, the training time of multiscale EM for fixed iteration numbers grew roughly exponentially with training sample size, while the training time of multiscale SID had minimal changes as the training sample size increased (figure 3(a)). Also, as expected, more EM iterations required increasingly more time to run (figure 3(a)). These results indicate that multiscale EM is increasingly more computationally expensive to train compared with multiscale SID for larger training sample sizes. Overall, multiscale SID was significantly faster in training time than multiscale EM across all simulated training sample sizes (FDR-corrected $P\unicode{x2A7D}2.40\times10^{-4}$, $N_\mathrm{s} = 50$). Note that training time in EM is the time it takes for it to converge, unless it is specified for a fixed number of iterations.

*Dynamical modes:* Second, we explored the accuracy in identifying the dynamical modes, which, as noted earlier, are the eigenvalues of the state transition matrix **A** and quantify the dynamics. For visualization, we show one example simulated model in figure 3(c) that illustrates the error between the true and identified eigenvalues from which the normalized mode error is computed per section 2.3.2 and how this error is decreased by increasing training sample size. We found that both the real and the imaginary parts of the mode error decreased by increasing the training sample size. Interestingly, multiscale SID was significantly more accurate than multiscale EM in identifying the dynamical modes (figures 3(d) and (e)). For $T = 5\times10^4$ training samples, the normalized mode error in multiscale SID was $0.60\pm0.04\%$ vs. $5.31\pm1.70\%$ for multiscale EM (figure 3(e), $P = 3.99\times10^{-8}$, $N_\mathrm{s} = 50$) and the medians for the two methods were $0.59\%$ vs. $1.20\%$, respectively. We note that while multiscale SID is significantly more accurate than multiscale EM in the mode identification here, both methods are accurate as evident by their low absolute normalized errors.

Even as we increased the training sample size, mode identification in multiscale SID was significantly more accurate than multiscale EM across all simulated training sample sizes (FDR-corrected $P\unicode{x2A7D}3.20\times10^{-3}$, $N_\mathrm{s} = 50$). Also, as expected, the normalized mode error monotonically decreased with increasing training sample size for both algorithms, and with more EM iterations for multiscale EM (figure 3(d)). The reasons for more accurate performance of multiscale SID in mode identification could be the approximations that must be made in multiscale EM to find the posterior density, the fact that EM aims to optimize the neural data likelihood rather than dynamic mode identification, and that multiscale EM does not guarantee to even optimize the neural data likelihood given its approximations [6, 94] (see section 4).

*Neural prediction:* Third, we compared the multiscale SID and multiscale EM in predicting the simulated multimodal neural activity (see section 2.3.4, figure 4). We found that despite being much faster (figure 3(b)), multiscale SID still had comparable performance to multiscale EM even in neural prediction when provided with enough training samples (figure 4). Note that multiscale SID was more accurate than multiscale EM in dynamical mode identification (figure 3(e)) but nevertheless they both had low mode errors in terms of the absolute normalized error values. For field potentials, this accuracy was quantified with CC between the predicted and true field potential activity and for spiking activity with PP (defined in section 2.3.4). With $T = 5\times10^4$ training samples, the one-step-ahead prediction accuracy of field potentials and spiking activity for multiscale SID and multiscale EM were within 0.38% and 1.7% of each other, respectively (figures 4(b) and (d)). Also, the prediction of neural activity monotonically improved with training sample size for both algorithms and with EM iterations for multiscale EM (figures 4(a) and (c)).

#### 3.1.4. Multiscale SID can fuse information across discrete and continuous neural modalities and identifies the dynamical modes better than single-scale SID

Multiscale modeling allows information across multiple neural modalities to be aggregated and thus has the potential to outperform modeling of any single modality in terms of learning the neural dynamics. To demonstrate this capability, we simulated 50 multiscale models with 14 discrete spiking signals, 14 continuous field potential signals and 4 dynamical modes that were a mixture of shared modes between the two modalities as well as exclusive modes to each modality (distinct modes). We then gradually combined increasingly more neural signals from one modality with a fixed number of signals from the other modality, referred to as the baseline signals (section 2.3.6). We used single-scale SID to identify a model for the baseline signals and used multiscale SID to do so for the concatenation of baseline signals and the signals from the second combined modality.

**Figure 5.**
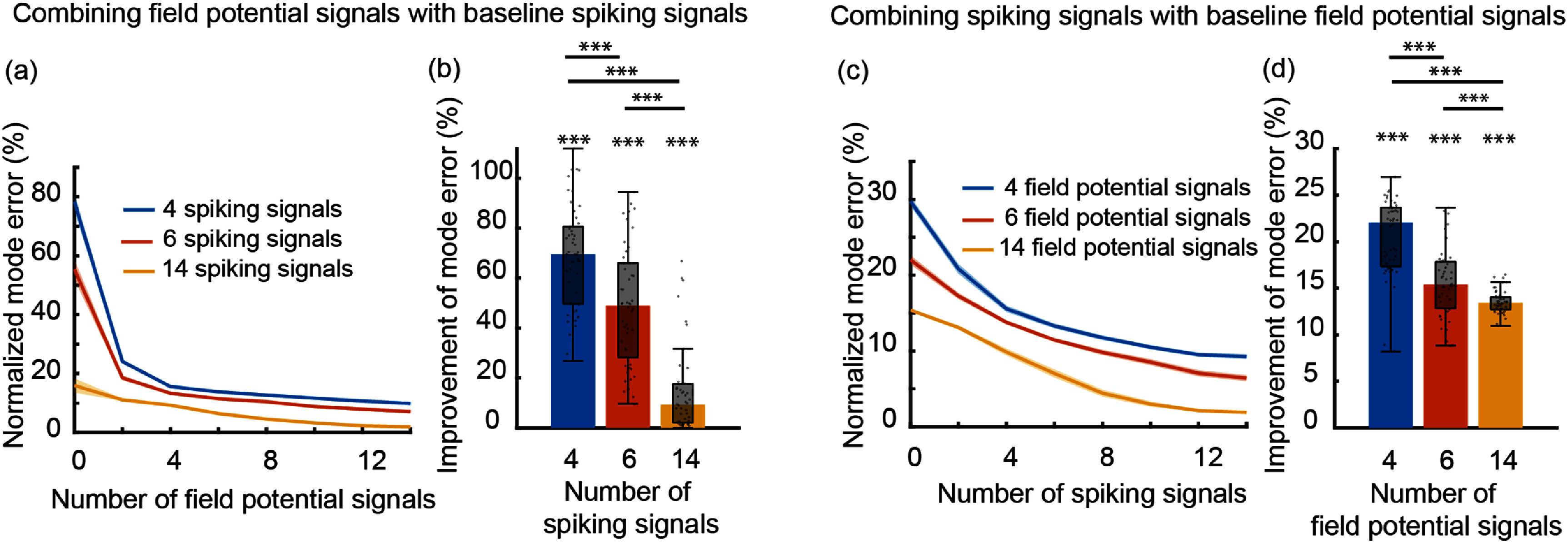
Multiscale SID outperforms single-scale SID in identification of dynamical modes in simulations due to fusion of information across modalities, with the largest performance gain being obtained in the low information regime. (a) Normalized mode error (section 2.3.2) as increasingly more continuous field potential signals are gradually combined with 4, 6, or 14 baseline discrete spiking signals. The start of the curves (i.e. 0 on *x*-axis) indicates normalized mode error for single-modal signals (i.e. spiking signals only). Solid line indicates mean across 50 simulated neural network systems and shaded area represents s.e.m ($N_\mathrm{s} = 50$ data points). (b) Comparison of the maximum improvement of normalized mode error after combining continuous field potential signals with 4, 6, or 14 baseline discrete spiking signals. Bars represent the median and box conventions are the same as in figure 3. Dots represent individual data points. Asterisks indicate significance of a pairwise comparison of improvement value across baseline regimes as well as comparison of improvement value with 0 (Wilcoxon signed rank test, $N_\mathrm{s} = 50$, ***: *P* < 0.0005). (c) and (d) Same as (a) and (b) but for gradually combining increasingly more spiking signals with a fixed number of baseline field potential signals.

We found that the learned models became increasingly more accurate as more and more signals of a second modality were combined with the signals from the first baseline modality (figures 5(a) and (c)). Specifically, the normalized mode error monotonically decreased as field potential signals were combined gradually with a fixed number of spiking signals or vice versa (figures 5(a) and (c)). These results suggest that multiscale SID can correctly aggregate information across discrete and continuous modalities.

We next compared the maximum improvement gained by going from single-scale to multiscale modeling for cases with different numbers of baseline signals (figures 5(b) and (d), section 2.3.6). We found that the improvement in identification error of dynamical modes was significantly larger than 0 (figures 5(b) and (d), FDR-corrected $P\unicode{x2A7D}1.23\times10^{-9}$, $N_\mathrm{s} = 50$), and was larger for the lower information regime, i.e. when the number of baseline signals was smaller (figures 5(b) and (d), FDR-corrected $P\unicode{x2A7D}1.58\times10^{-4}$, $N_\mathrm{s} = 50$). This result suggests that for learning the dynamics, multiscale modeling has the most benefit compared with single-scale modeling in the low information regime, i.e. when the initial modality has incomplete information about the neural dynamics.

**Figure A2.**
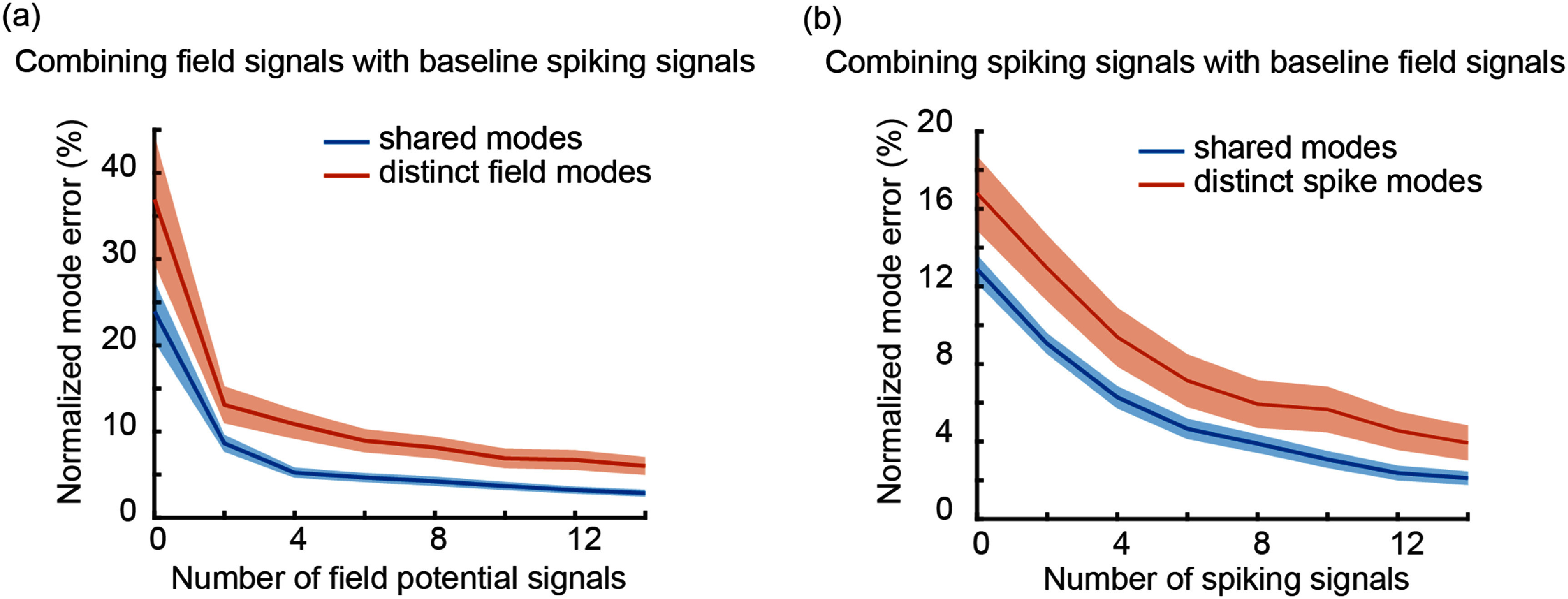
Multiscale SID (compared to single-scale SID) improves the identification of collective dynamics in both modalities. (a) Normalized errors of shared modes (blue) and distinct field modes (orange) that are only present in the field potential activity, as increasingly more field potential signals combined with six spiking signals. The start of the curves (i.e. 0 on *x*-axis) indicate normalized mode error for single-modal signals (i.e. spiking signals only). Solid line indicates mean across 50 simulated neural network activities and shaded area represents s.e.m. (b) same as (a) but for combining increasingly more spiking signals with six field potential signals. These simulation results suggest that multiscale SID correctly combines information across modalities of neural activity.

As mentioned earlier, in these numerical simulations and motivated by previous studies [49], we simultaneously simulated modes that were shared between the two modalities as well as modes that were exclusive to each of the modalities (distinct modes) (see sections 2.3.1 and 2.3.6). We found that combining one modality with another improved the learning of both the modes that were exclusive to the added modality as well as the modes that were shared between the two modalities. This result was found by analyzing the identification error of distinct and shared modes separately (figure A2), and again shows that information is being aggregated across modalities about their collective dynamics to learn them more accurately.

### 3.2. Multiscale SID accurately predicts the spike-LFP recordings from the NHP brain during naturalistic movements, while being substantially faster

**Figure 6.**
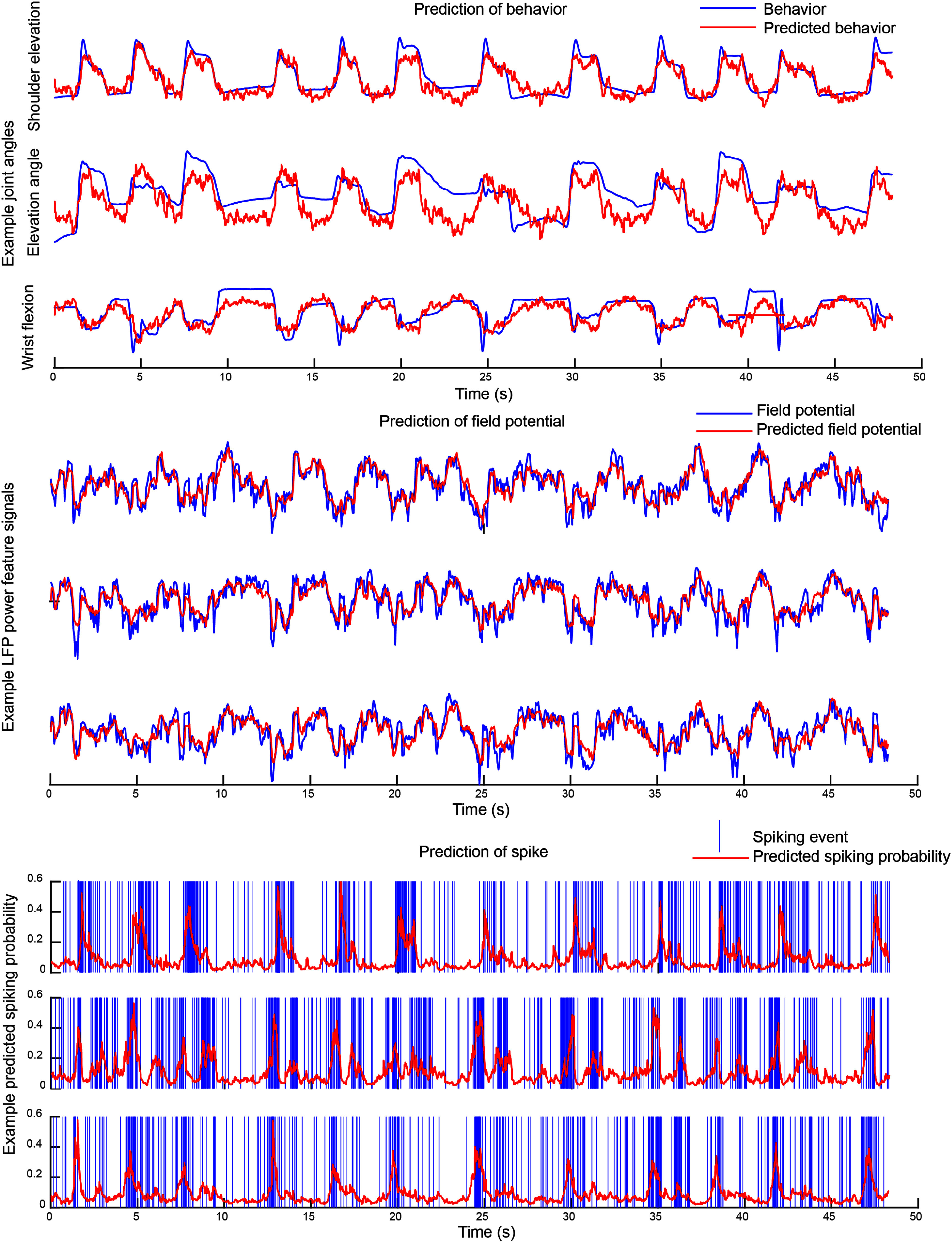
Prediction of example behavior trajectories, field potential and spiking activity in one test fold of NHP datasets. We learn the multiscale model using multiscale SID with $n_x = 24$ in the training data and use it in the test data to extract the latent states and predict behavior and spike-LFP activity from these states (see sections 2.3.4, 2.4.3 and 2.4.4). (a) Example joint angle time-series and their predictions. (b) Example field potential power feature time-series and their one-step-ahead predictions. (c) Example spike events and their one-step-ahead predicted probability (probability of having at least one spiking event in the 10 ms time bin). This predicted probability is expected to be higher when more spiking events are occurring. Each vertical blue line indicates the time of one spike event.

**Figure 7.**
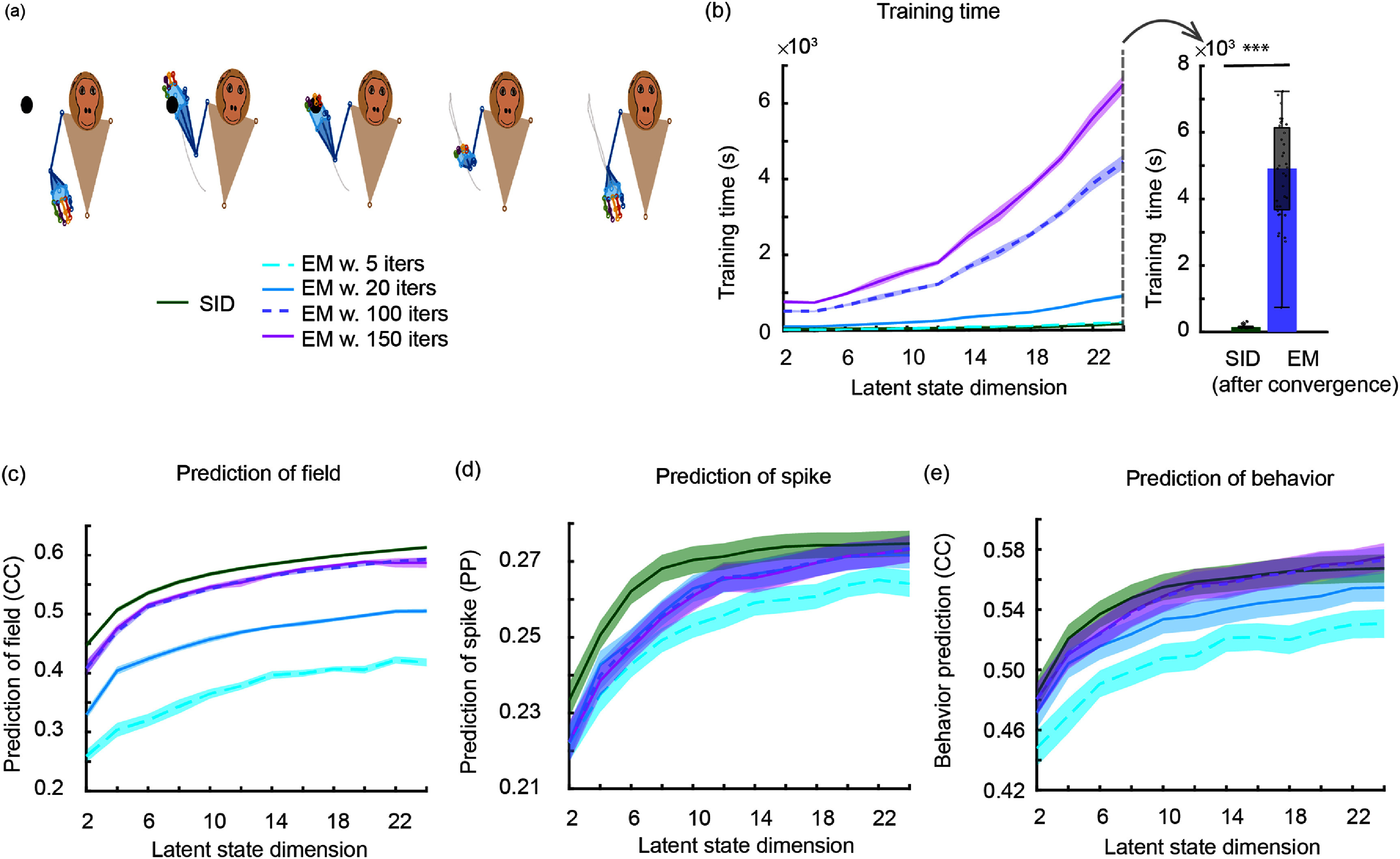
In NHP datasets, multiscale SID outperforms multiscale EM in both training time and neural prediction accuracy and matches the accuracy of multiscale EM in prediction of behavior. (a) Spike-LFP activity was recorded as a non-human primate performed naturalistic reach and grasp movements to randomly positioned objects in 3D space. (b) Left: training time (in seconds) of multiscale SID and multiscale EM with different number of iterations as a function of latent state dimension. Training time monotonically increases with more multiscale EM iterations or latent state dimensions. Solid line represents the mean and shaded area represents s.e.m. over folds and data sessions ($N_\mathrm{s} = 35$ data points). Right: comparison of training time of multiscale SID and that of multiscale EM at $n_x = 24$. Multiscale SID is significantly faster, approximately 30 times faster than multiscale EM. Bar, box and asterisks conventions are as in figure 3. (c) and (d) Same as left panel of (b) but for one-step-ahead prediction accuracy of field potentials (quantified by correlation coefficient (CC), section 2.3.4), one-step ahead prediction accuracy of spiking activity (quantified by prediction power (PP), section 2.3.4), and behavior prediction accuracy (quantified by CC, section 2.4.3).

We next used multiscale SID to model multimodal spiking and LFP activity recorded from an NHP while performing a naturalistic 3D reach and grasp movement task (figure 7(a), see section 2.4.1 and [23, 49, 78] for more details). We obtained discrete spiking activity **N**_*t*_ by detecting the threshold crossing events every 10 ms and field potential activity **y**_*t*_ by computing power features in seven standard frequency bands from the recorded neural signals every 50 ms (see section 2.4.2). During model learning alone, we interpolate the power features to recover the samples of **y**_*t*_ at every 10 ms time-step that spike counts are observed; note, during inference, no interpolation is necessary and these intermediate field samples can simply be treated as missing observations in an MSF [43] (see sections 2.2.2, 2.2.3 and 2.3.4). We used a five-fold cross-validation scheme. We learned the multiscale model using multiscale SID in the training data and then used it in the test data to extract the latent states and predict the spike-LFP activity and behavior (i.e. joint angles) from the extracted latent states (see sections 2.3.4, 2.4.3 and 2.4.4). Example spike-LFP and behavior time-series along with their predictions using multiscale SID with $n_x = 24$ are shown in figure 6. We also compared the multiscale SID with the existing multiscale EM for spike-LFP data and with single-scale SID for spikes alone or LFP alone (sections 2.4.5 and 2.4.6). For each method, we fitted models with latent state dimensions spanning $n_x\in\{2,4,{\ldots},24\}$ (section 2.4.5). Convergence criteria for EM was set based on convergence of neural prediction performance as described in section 2.4.5, with maximum allowed EM iterations of 150.

#### 3.2.1. Multiscale SID outperforms multiscale EM in training time and spike-LFP prediction in the NHP dataset

We found that the training time of multiscale SID was significantly faster than that of multiscale EM across all latent state dimensions (figure 7(b) left panel, FDR-corrected *P*-value $\unicode{x2A7D} 1.29\times10^{-7}$, ${N}_\mathrm{s} = 35$). Interestingly, in addition to being faster, multiscale SID was also significantly more accurate in prediction of LFP across all latent state dimensions and in prediction of spiking activity across latent state dimensions up to 22 (figures 7(c) and (d), FDR-corrected *P*-value $\unicode{x2A7D}1.70\times10^{-2}$, ${N}_\mathrm{s} = 35$). For example, for the maximum considered state dimension of $n_x = 24$, multiscale SID was $30.66\pm1.84$ times faster than multiscale EM while reaching 3.5% more accurate LFP prediction CC and slightly more accurate spike prediction PPs (figure 7). Thus, in addition to its substantially lower training time, multiscale SID could outperform or do comparably to multiscale EM.

Finally, we computed the accuracy in predicting behavior as quantified by the CC between the predicted and true joint angle trajectories (see section 2.4.3). We found that despite its much faster training time, multiscale SID had similar accuracy in predicting behavior compared to multiscale EM (figure 7(e)), and that it took multiscale EM substantially longer training time to achieve this accuracy. The substantially longer training time in multiscale EM is due to its iterative and computationally expensive nature, and meant that multiscale SID was able to reach better or comparable modeling accuracy using much faster computations (figure 7(b)).

#### 3.2.2. Contribution of modes to spiking activity, LFP activity, and behavior

**Figure A3.**
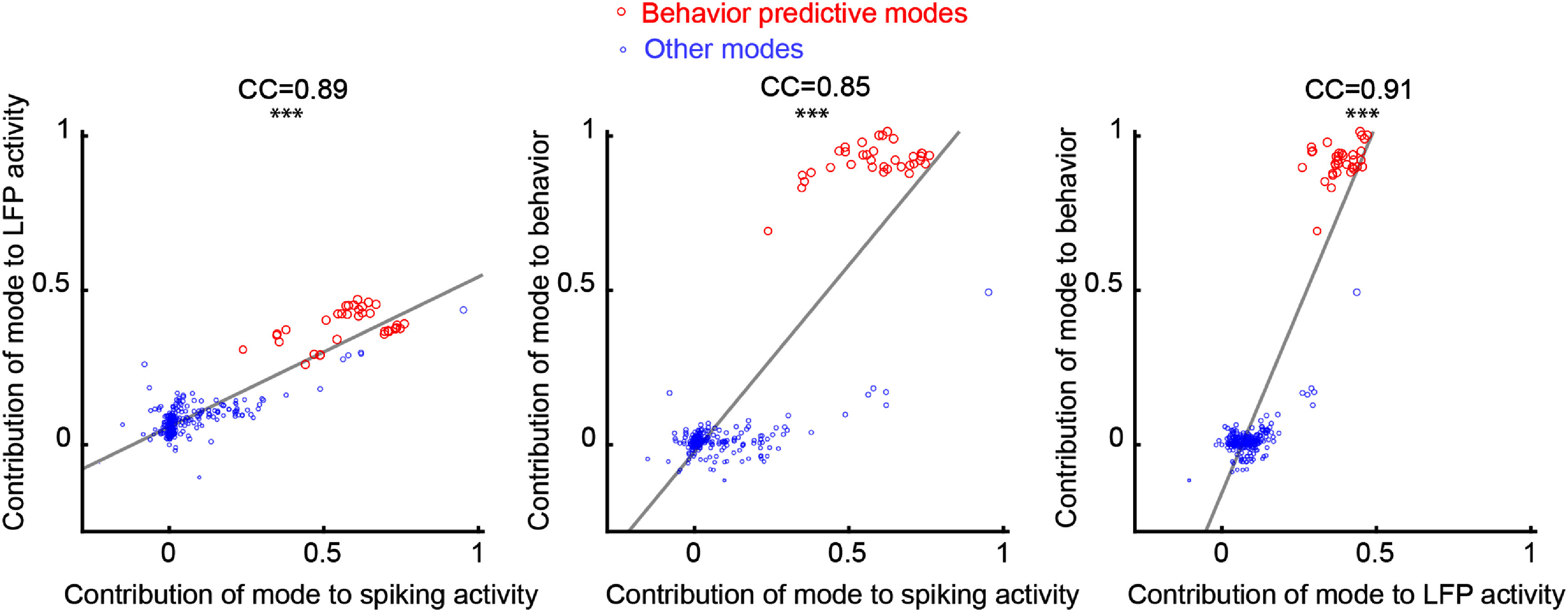
The contribution of dynamical modes to LFP activity, spiking activity, and behavior and their correlation quantified by Pearson correlation coefficient (CC). The behavior predictive modes are shown by red dots. Dots correspond to modes identified by multiscale SID using latent state dimension $n_x = 14$ in 5 folds and 7 data sessions, and the dot sizes are proportional to the mode contributions to behavior prediction. The grey line is the least squares fitted line to the dots in each panel. Asterisks indicate significance of Pearson’s CC between contributions of modes in different modalities ($N_\mathrm{s}$ = 312, ***: *P* < 0.0005).

To investigate the relationship between the dynamics of spiking and LFP modalities in the NHP datasets, we assessed the contribution of each dynamical mode (identified by multiscale SID) to each neural modality and to behavior (see appendix J). Across sessions and folds, the contribution of modes to spiking activity was significantly correlated with their contribution to LFP activity; further, the contribution of modes to behavior was significantly correlated to their contribution to each of the neural modalities (figure A3, $N_\mathrm{s} = 312$, $\mathrm{CC}\gt0.855$, Pearson’s correlation $P\lt8.66\times10^{-91}$, $n_x = 14$). Looking at these results more closely, we found that behavior predictive modes were strongly contributing to both spiking and LFP activity, suggesting that they are shared between spiking and LFP activity—these modes are shown by the red dots in figure A3 and indicate the modes with the largest contribution to behavior in each fold. In addition to these shared modes, we also found modes in the left panel of figure A3 that were strongly present in one neural modality but weakly present in the other neural modality, suggesting that distinct modes also exist in these two modalities. Taken together, these results suggest that both shared and distinct modes exist in spiking and LFP signals, and that the behavior predictive modes are shared between the two modalities as also suggested in prior work [49].

#### 3.2.3. Multiscale SID improved behavior decoding in the NHP dataset compared with single-scale SID due to addition of spike-LFP information

We next performed a neural signal combination analysis similar to what we performed for simulated data (sections 2.4.6, 2.3.6 and 3.1.4). We constructed a pool of 30 spike channels and 30 LFP channels and gradually combined increasingly more signals from one modality with a fixed number of signals from the other modality, referred to as the baseline neural signals (section 2.4.6). We identified a model for the baseline neural signals on their own using the single-scale SID and learned models for the multimodal signals (baseline plus added signals) using the multiscale SID. We computed the cross-validated behavior prediction accuracy for each learned model.

**Figure 8.**
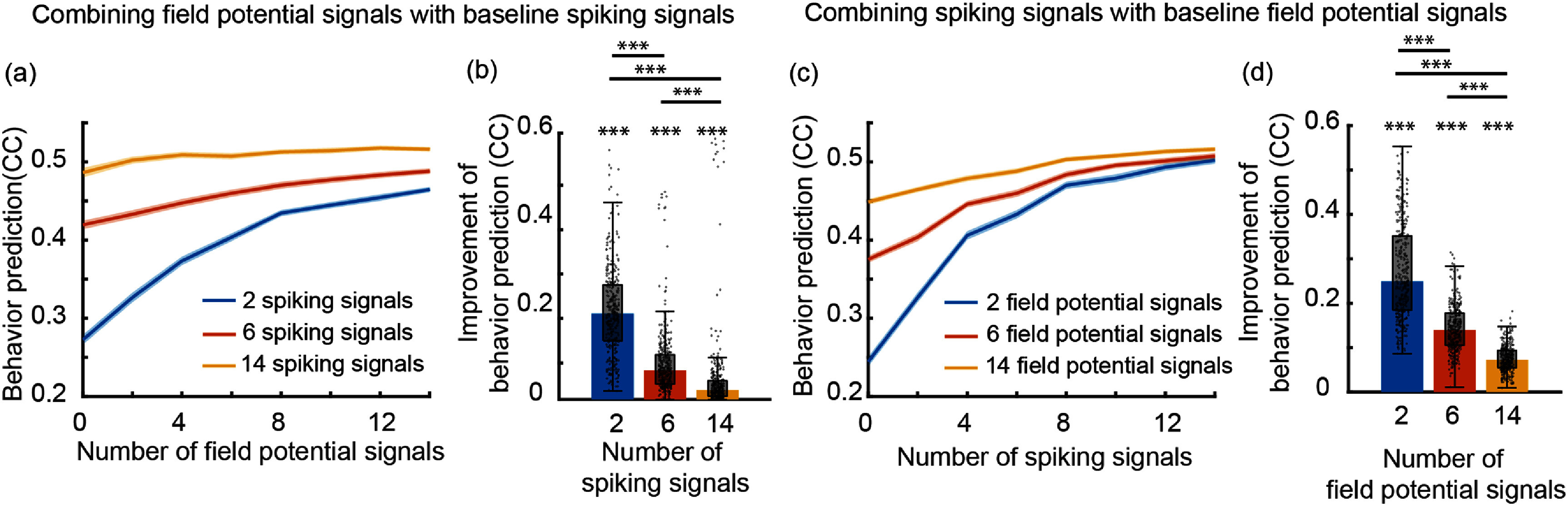
In NHP datasets, multiscale SID improves the behavior prediction accuracy compared to single-scale SID due to fusion of information across modalities and this improvement is largest in the low information regime. (a) Behavior prediction accuracy quantified by correlation coefficient (CC) between the predicted and true behavior as increasingly more field potential signals are combined with to 2 (blue), 6 (red) and 14 (yellow) baseline spiking signals. The start of the curves (i.e. 0 on *x*-axis) indicates behavior prediction for single-modal neural activity (i.e. spiking signals only). Solid line indicates mean across folds, random sets of selected neural signals and data sessions and shaded area represents s.e.m ($N_\mathrm{s} = 350$ data points). (b) Comparison of the maximum improvement of behavior prediction (quantified by difference of CCs) after combining field potential signals with 2 (blue), 6 (red) and 14 (yellow) spiking signals (section 2.4.6). Bars indicate median and box conventions are as in figure 3. Dots represent individual data points. Asterisks indicate significance of a pairwise comparison of improvement value across baseline regimes as well as comparison of improvement value with 0 (Wilcoxon signed rank test, $N_\mathrm{s} = 350$, ***: *P* < 0.0005). (c) and (d) Same as (a) and (b) but for combining increasingly more spiking signals with a fixed number field potential signals (baseline field potential signals).

We found that behavior prediction performance benefited from multiscale modeling (figure 8), and monotonically improved both as increasingly more field potential signals were gradually combined with baseline spiking signals and vice versa (figures 8(a) and (c)). Further, similar to our simulation results, the maximum improvement in behavior prediction performance using multiscale SID was significantly larger than 0 (figures 8(b) and (d), FDR-corrected $P\unicode{x2A7D}6.77\times10^{-51}$, $N_\mathrm{s} = 350$), and was larger for the lower information regime, i.e. when the number of baseline signals was smaller (figures 8(b) and (d), FDR-corrected $P\unicode{x2A7D}1.33\times10^{-30}$, $N_\mathrm{s} = 350$). Note that if we keep increasing the number of baseline signals, this improvement may become marginal and even insignificant as the behavior decoding may already become saturated using the baseline signals. For example, the median of improvement for 20 baseline field potential signals was only $4.45\%$ and for 20 baseline spiking signals was $1.32\%$, although these improvements still remained significantly greater than 0. Also, note that this improvement was obtained regardless of whether the baseline modality was the discrete spiking or the continuous LFP modality. This bidirectional improvement suggests that the advantage of multiscale over single-scale modeling was not simply due to the dominance of one modality over the other, but rather due to the addition of information across them. Together, these results suggest that for NHP multimodal spike-LFP data, multiscale SID is correctly aggregating information across neural modalities.

## 4. Discussion

We developed multiscale SID, an analytical method that efficiently learns multiscale dynamical models of multimodal discrete-continuous spike-field population activity, extracts their low-dimensional latent dynamics, and enables causal and multimodal statistical inference of latent states, as well as prediction of neural activity and behavior. We validated this method using extensive numerical simulations and NHP spike-LFP activity recorded during 3D naturalistic reach and grasp movements [49, 78]. Multiscale SID accurately learned the multiscale model parameters and the low dimensional dynamical modes in spike-field population activity. Also, it fused information across spiking and field potential modalities, thus more accurately identifying the dynamical modes and predicting the behavior compared to when using either modality alone. Moreover, multiscale SID had a much lower training time compared to the existing multiscale EM method, while being better in identifying the dynamical modes and having a better or similar accuracy in predicting neural activity and behavior. These capabilities are important in studying multimodal neural dynamics and developing multimodal neurotechnology especially for real-time and adaptive experiments where training time may be a limiting factor. Finally, while we focused on modeling multimodal spike-field dynamics, multiscale SID provides a general analytical method that can be applied broadly to other multimodal discrete-continuous time-series.

### 4.1. Flexible approach to find valid noise statistics based on the model and inference structures

One of the key challenges in developing multiscale SID was that noise statistics in the multiscale SID model (equation (1)) must satisfy certain conditions including being PSD. These conditions are important not only for accurate modeling, but also, critically, for enabling statistical inference of the latent states and behavior. While there are traditional non-covariance-based SID methods that can at least guarantee PSD conditions for covariance matrices [70, 71], these algorithms are not applicable for the multiscale model (equations (1) and (2)). This is because these methods require direct access to continuous observations—and not just their covariances—, but log firing rates (**z**_*t*_) are not observable and their corresponding observable spike counts are not continuous (see section 2.2.2). Thus, after computing the cross-covariances, we had to utilize a covariance-based SID approach, which does not guarantee valid noise statistics as is known in the literature [70]. To address this challenge, we introduced a novel approach where we devised a constrained optimization problem to learn valid noise statistics (section 2.2.6).

Beyond the positive semi-definiteness of noise covariances, this flexible constrained optimization approach further allowed us to incorporate other conditions needed by the multiscale model to derive the currently established multiscale inference algorithm, i.e. MSF [43] (e.g. $\mathbf{R}_z = 0$ in equation (2)). Flexibly imposing such additional constraints is not addressed in current covariance-based or non-covariance-based SID methods [70–72]. Note, however, that unlike the positive semi-definiteness, these other conditions are not fundamental requirements of the model, but rather engineering design choices made in prior work to make the derivation of a causal multiscale inference method tractable (without these conditions, currently no such inference method is available). Our novel constrained optimization approach could also be used by future work in other settings involving SID methods and their extensions, for example in developing SID for other observation distributions or to impose alternative constraints on noise statistics learned by SID.

### 4.2. Comparison of multiscale SID with EM in training time and accuracy

While multiscale EM learns a set of parameters for the same model structure, our results in simulations and in real data show that multiscale SID has much lower training time while achieving better dynamical mode identification and better or similar neural prediction. Moreover, this advantage gets more pronounced as the training sample size increases as shown in figures 3(a) and (d). There could be multiple reasons for this.

In terms of computation cost, the higher training time of multiscale EM is because of its iterative numerical nature. At each iteration, multiscale EM needs to run the expectation step which involves filtering and smoothing the entire training data [43, 48] and the maximization step, which requires solving an optimization problem [48]. In contrast, multiscale SID largely consists of a specific set of non-iterative analytical algebraic operations.

In terms of accuracy, our results show that multiscale SID learns the dynamical modes more accurately than multiscale EM, and performs better or similarly for neural prediction in real neural data or in simulations when provided with enough training samples (figures 3(d), (e), 4 and 7(c), (d)). The better performance of multiscale SID could be due to the approximations that multiscale EM has to make to find the posterior density. In particular, multiscale EM uses the Laplace approximation to approximate the posterior density with a Gaussian distribution, just as is done in single-scale EM for Poisson observations [4, 61]. Given that the Laplace approximation may fail to capture broader statistics of the true posterior distribution [95], it may lead to suboptimal parameter identification and neural predictions. Furthermore, given the Laplace approximation, there is no guarantee for non-decreasing data likelihoods in consecutive iterations, which is the objective of EM [6, 94]. Overall, the higher efficiency of multiscale SID compared with multiscale EM in training time while maintaining better or similar accuracy can make it beneficial for multiscale modeling especially when efficient learning is desired or needed.

Finally, it is worth noting that beyond EM, other numerical techniques can also be computationally expensive in training and in many cases may not enable causal statistical inference. For example, prior works have developed non-causal numerical variational inference methods for continuous functional magnetic resonance imaging (fMRI) and categorical behavioral data [96]. But these techniques did not focus on spike-field neural data. Moreover, similar to EM, these methods can have a high computational cost in training compared with SID given their iterative numerical approach, which is burdensome especially for real-time or adaptive learning. Finally and critically, enabling real-time applications requires the ability for causal inference/decoding, which is not achieved by these variational inference methods [96]. The new multiscale SID method addresses all these challenges.

### 4.3. Multiscale SID for other multimodal distributions

Here we derived the multiscale SID for joint modeling of continuous Gaussian and discrete Poisson modalities (equation (1)). However, the multiscale SID framework can flexibly generalize to other multimodal distributions as long as they conform to the GLMs. This can be done because the moment transformation step for empirical estimation of covariances **H**_*w*_ is flexible and easily extendable to other GLMs, and because the other steps of the derivation do not depend on the distribution of observations, and the constrained optimization here can flexibly enforce assumptions needed for different observation distributions. Also, while our demonstrations in real data were for spike-LFP recordings, future work can apply the multiscale SID for modeling of spikes along with other continuous neural modalities such as intracranial EEG and electrocorticogram (ECoG). More broadly, beyond neural data, multiscale SID can also be applied to other multimodal discrete-continuous time-series to model them collectively.

### 4.4. Limitations of multiscale SID

The multiscale model here imposes the assumptions in prior work that made deriving an MSF tractable (e.g. $\mathbf{R}_z = 0$ in equation (2)) but that may not be met by data. Developing novel causal MSFs that do not require these assumptions may help with model accuracy but could be challenging in terms of derivation/tractability. Further, the multiscale model assumes that state dynamics are linear. Linear dynamical models have had much success for both neuroscience investigations and BMIs given their nice properties such as interpretability and real-time inference capability [11, 26, 65, 66, 74, 97, 98]. Also, given enough latent state dimension, linear dynamical models have been shown to describe neural dynamics well [24, 75, 99]. Nevertheless, neural dynamics could be nonlinear and thus future work can explore developing learning methods for nonlinear multiscale dynamics that also enable causal inference/decoding.

### 4.5. Information fusion across neural modalities

Previous studies of spiking and LFP have mostly focused on quantifying the amount of task related information in each modality, rather than studying their low dimensional state dynamics and how these dynamics relate across modalities [30, 31, 33, 34, 36, 39, 41–44]. Some of these studies find similar amount of task related information in these modalities, while others suggest that there exists non-redundant information in each modality. For example, some studies found that spiking and LFP achieve similar decoding of the direction of saccades and reaches in the posterior parietal cortex [30, 33]. Others found that hand speed is encoded better in LFP, while its position is encoded better in the spiking activity in the motor cortex [42]. We recently studied the low dimensional state dynamics of spiking and LFP in the motor cortical areas during naturalistic reach-and-grasp movements [49]. We found a mode that was at a similar (shared) location in the eigenvalue space in spiking and LFP activities and dominantly predicted behavior. In this study, we expanded on these analyses by quantifying the actual contribution of the identified multiscale modes to the spiking and LFP modalities (section 3.2.2 and appendix J). We found that behavior predictive modes are largely the ones that strongly contribute to the dynamics in both spiking and LFP activity, suggesting that these behavior predictive modes are shared between the two modalities. This result is thus also consistent with the shared location of modes in the eigenvalue space found in spiking and LFP activity in our previous study [49] (figure A3, section 3.2.2). Furthermore, we also found modes that strongly contributed to one modality while weakly contributing to the other one (figure A3), suggesting that there also exist distinct modes in each modality.

Given the possibility of both shared and distinct information in different neural modalities, allowing for multimodal modeling can not only improve the decoding of shared information, but also allow for decoding of distinct information that may not be possible with a single modality. This capability is enabled because multiscale SID learns not only the dynamics that are shared across the modalities, but also dynamics that are distinct to either modality, i.e. the collective dynamics of both modalities (figures 5 and A2). Consequently, multimodal modeling can also make future neurotechnologies more robust to neural signal loss. For example, spiking activity in chronically implanted electrodes may degrade over time faster than field potential modalities such as LFP or ECoG [41, 44, 58, 100]. Thus, combining spikes with field potentials can help in mitigating the impact of such degradation on identifying the shared dynamics, consistent with our simulation results (figure A2). As behavior predictive modes in the NHP dataset were largely shared between spiking and LFP (figure A3), adding LFP may lead to more robust behavior decoding over time.

### 4.6. Applications and future research directions

Given its computational efficiency in training, the multiscale SID method can enable various future real-time learning applications in neuroscience and neural engineering. Tracking non-stationarity in neural representations is a major challenge in BMIs, which can happen due to various factors such as recording instability [101–103], learning and plasticity [58, 67, 98, 104–114], or a change in an internal state such as a psychiatric state [115]. These non-stationarities need to be addressed by relearning or updating the model parameters frequently, even potentially continuously at every time step [67]. Indeed, prior BMI works have shown that recalibration is critical to performance [63–66, 68] and that continuous adaptation/recalibration at a faster rate (e.g. every time step) could allow for faster and more accurate parameter convergence during closed-loop model training [63, 64]. EM can make such closed-loop adaptation/recalibration impractical given its substantial training time/complexity. Beyond adaptation, these applications may also face practical difficulties for deployment in a given session using EM due to extended offline training time.

Future work can utilize the multiscale SID method to develop an adaptive/online learning algorithm for tracking plasticity and non-stationarities [22, 25] in multimodal neural signals, which updates/relearns the model parameters in real-time. We recently developed an SID-based adaptive learning algorithm for single-scale continuous neural activity [25] and demonstrated its success in tracking of non-stationarity in multi-day ECoG recordings from epilepsy patients [22]. Developing a multiscale adaptive learning algorithm will be an important direction for future investigation. Adaptive tracking of neural dynamics is especially important for studying plasticity in the brain [116, 117] and for neurotechnologies that need to operate over long time periods, such as closed-loop deep brain stimulation systems [97, 118–123] or BMIs [97, 124–132].

Our results suggest that multiscale SID can be accurate for various applications in BMIs and neuroscience for two reasons. First, prior BMIs have achieved high performance even using just spiking activity [63–66], for example using the point process filter compared to here [63, 64]; we show that the multiscale SID and its associated MSF outperform this spike decoding by successfully fusing information across modalities. Second, for learning, EM is widely used in neuroscience [2, 4, 6, 11, 49, 61, 87, 133, 134]; we show that for multiscale data, multiscale SID performs similarly or somewhat better than multiscale EM.

Recent work have shown the benefit of learning a dynamical model for neural-behavioral data together by developing an SID-based method for two signal sources termed preferential SID or PSID [23]. Compared to modeling of neural dynamics unsupervised with respect to behavior, PSID preferentially learns the behaviorally relevant neural dynamics, thus achieving better neural decoding of behavior using lower-dimensional latent states [23]. But PSID models a single modality of neural activity so far. Thus, extending the multiscale SID method to consider multimodal neural data together with behavioral data during learning could be an interesting future direction. This could allow us to preferentially learn the multimodal neural dynamics that are behaviorally relevant and dissociate them from behaviorally irrelevant dynamics, thus potentially learning the former more accurately.

## Data Availability

The main data supporting the results are available within the paper. The raw data is too large to be hosted and shared publicly. The data that support the findings of this study are available upon reasonable request from the authors.

## Code availability statement

The code used to support the results is available at https://github.com/ShanechiLab/MultiscaleSID.

## Appendix A Empirical moment computation

To empirically compute the moments *µ* ^*y*^ and $\Sigma^{yy}$, as defined in equation (17), we concatenate future–past vectors $\mathbf{y}^\mathrm{fp}_{t}\in\mathbb{R}^{2h_yn_y\times 1}$ (equation (16)) across available time steps in the training data and denote it as $\mathbf{y}^\mathrm{fp}\in\mathbb{R}^{2h_yny\times T-2h_y+1}$:

\begin{align*} \mathbf{y}^\mathrm{fp}\triangleq \left(\begin{array}{c}\mathbf{y}^\mathrm{f}\\ \hline\mathbf{y}^\mathrm{p}\end{array}\right)\triangleq \left( \begin{array}{cccc} \mathbf{y}_{h_y+1}&amp;\mathbf{y}_{h_y+2}&amp;\cdots&amp; \mathbf{y}_{T-h_y+1} \\[4pt] \mathbf{y}_{h_y+2}&amp;\mathbf{y}_{h_y+3}&amp;\cdots&amp; \mathbf{y}_{T-h_y+2} \\[4pt] \vdots&amp;\vdots&amp;\vdots&amp;\vdots\\ \mathbf{y}_{2h_y}&amp;\mathbf{y}_{2h_y+1}&amp;\cdots&amp; \mathbf{y}_{T}\\[4pt] \hline \mathbf{y}_{h_y}&amp;\mathbf{y}_{h_y+1}&amp;\cdots&amp; \mathbf{y}_{T-h_y} \\[4pt] \mathbf{y}_{h_y-1}&amp;\mathbf{y}_{h_y}&amp;\cdots&amp; \mathbf{y}_{T-h_y-1} \\[4pt] \vdots&amp;\vdots&amp;\vdots&amp;\vdots\\[4pt] \mathbf{y}_{1}&amp;\mathbf{y}_{2}&amp;\cdots&amp; \mathbf{y}_{T-2h_y+1}\\ \end{array} \right). \end{align*}

Then, *µ* ^*y*^ is the sample mean of each row of $\mathbf{y}^\mathrm{fp}$ and

\begin{equation*} \Sigma^{yy} = \left(\mathbf{y}^\mathrm{fp}-\mu^{y}\right)\left(\mathbf{y}^\mathrm{fp}-\mu^{y}\right)^{^{\prime}}/\left(T-2hy\right). \end{equation*}

Similarly, we concatenate $\mathbf{N}^\mathrm{fp}_{t}$ across available time steps in the training data and denote it as $\mathbf{N}^\mathrm{fp}\in\mathbb{R}^{2h_znz\times T-2h_z+1}$. We then empirically compute *µ* ^*N*^, $\Sigma^{NN}$, and $\Sigma^{Ny}$ as defined in equation (17) based on $\mathbf{N}^\mathrm{fp}$ and $\mathbf{y}^\mathrm{fp}$.

## Appendix B Transformation of moments for estimating $\Sigma^{zy}$

Our goal is to compute $\Sigma^{zy} = \mathrm{Cov}[\mathbf{z}^\mathrm{fp},\mathbf{y}^\mathrm{fp}]$ (section 2.2.3) in terms of first and second moments of spiking and field potential observation $\{\Sigma^{Ny},\Sigma^{NN},\Sigma^{yy},\mu^{N},\mu^{y}\}$, which are directly computable from data.

We have:

\begin{align*} \begin{array}{lcl} \mathbb{E}\left[\mathbf{N}^\mathrm{fp}_i\mathbf{y}^\mathrm{fp}_j\right]\!\!\!\!\!\!&amp; = &amp;\!\!\!\!\!\!\mathbb{E}\left[\mathbb{E}\left[\mathbf{N}^\mathrm{fp}_{i}\mathbf{y}^\mathrm{fp}_{j}|\mathbf{z}^\mathrm{fp}_i,\mathbf{y}^\mathrm{fp}_j\right]\right]\\[6pt] \!\!\!\!\!\!&amp; = &amp;\!\!\!\!\!\!\mathbb{E}\left[\mathrm{exp}\left(\mathbf{z}^\mathrm{fp}_{i}\right)\mathbf{y}^\mathrm{fp}_{j}\right] \end{array} \end{align*}

where ._*i*_ and ._*j*_ denote the *i*th and *j*th element of $\mathbf{N}^\mathrm{fp}_{t}$ and $\mathbf{y}^\mathrm{fp}_{t}$, defined in section 2.2.3. Given that $\mathbf{z}^\mathrm{fp}_{i}$ and $\mathbf{y}^\mathrm{fp}_{j}$ are jointly normal, i.e.:

\begin{equation*} \left(\begin{array}{c}\mathbf{z}^\mathrm{fp}_{i}\\ \mathbf{y}^\mathrm{fp}_{j}\end{array}\right)\sim \mathcal{N}\left( \left(\begin{array}{c} \mu^{z}_{i}\\[3pt] \mu^{y}_{j} \end{array}\right), \left(\begin{array}{cc} \Sigma^{zz}_{i,i}&amp;\Sigma^{zy}_{i,j}\\[3pt]\Sigma^{zy}_{i,j}&amp;\Sigma^{yy}_{j,j} \end{array}\right)\right), \end{equation*}

we can compute $\mathbb{E}[\mathrm{exp}(\mathbf{z}_{i})\mathbf{y}_j]$ in equation (47) as:

\begin{align*} \mathbb{E}\left[\mathrm{exp}\left(\mathbf{z}^\mathrm{fp}_{i}\right)\mathbf{y}^\mathrm{fp}_{j}\right] = \mathrm{exp}\left(\frac{1}{2}\Sigma^{zz}_{i,i}+\mu^{z}_{i}\right)\left(\mu^{y}_{j}+\Sigma^{zy}_{i,j}\right) \end{align*}

by taking a double integral required to compute $\mathbb{E}[\mathrm{exp}(\mathbf{z}_{i})\mathbf{y}_j]$ with respect to the above bivariate normal distribution. Further, we have:

\begin{align*} \mu^{N}_{i}&amp; = \mathbb{E}\left(\mathbf{N}^\mathrm{fp}_{i}\right)\\[6pt] ~&amp; = \mathbb{E}\left[\mathbb{E}\left[\mathbf{N}^\mathrm{fp}_{i}|\mathbf{z}^\mathrm{fp}_i\right]\right]\\[6pt] ~&amp; = \mathbb{E}\left[\mathrm{exp}\left(\mathbf{z}_{i}^\mathrm{fp}\right)\right]\\[6pt] ~&amp; = \mathrm{exp}\left(\frac{1}{2}\Sigma^{zz}_{i,i}+\mu^{z}_{i}\right). \end{align*}

According to equations (47)–(49), we have:

\begin{align*} \Sigma^{Ny}_{i,j}&amp; = \mathbb{E}\left[\mathbf{N}^\mathrm{fp}_i\mathbf{y}^\mathrm{fp}_j\right]-\mu_{i}^{N}\mu_{j}^{y}\\[6pt] &amp; = \mathbb{E}\left[\mathrm{exp}\left(\mathbf{z}^\mathrm{fp}_{i}\right)\mathbf{y}_j\right]-\mu_{i}^{N}\mu_{j}^{y}\\[6pt] ~&amp; = \mathrm{exp}\left(\frac{1}{2}\Sigma^{zz}_{i,i}+\mu^{z}_{i}\right)\left(\mu^{y}_{j}+\Sigma^{zy}_{i,j}\right)-\mu_{i}^{N}\mu_{j}^{y}\\[6pt] ~&amp; = \mu_{i}^{N}\left(\mu^{y}_{j}+\Sigma^{zy}_{i,j}\right)-\mu_{i}^{N}\mu_{j}^{y}\\ ~&amp; = \mu_{i}^{N}\Sigma^{zy}_{i,j}. \end{align*}

We then solve for $\Sigma^{zy}_{i,j}$ in equation (50), which gives:

\begin{align*} \Sigma^{zy}_{i,j} = \frac{\Sigma^{Ny}_{i,j}}{\mu^{N}_{i}}. \end{align*}

Of note, interestingly, the final relation in equation (51) resembles what [72] derives in their supplemental equation (6) as the moment transformation for relating Poisson observation to Gaussian *inputs*, as opposed to relating them to simultaneous Gaussian *observations*, which is derived here.

## Appendix C Constructing the future–past cross-covariance matrix **H**_*w*_ and auto-covariance matrix Λ_0_

Having estimated $\Sigma^{zz} = \mathrm{Cov}[\mathbf{z}_t^\mathrm{fp}]$, $\Sigma^{zy} = \mathrm{Cov}[\mathbf{z}_t^\mathrm{fp},\mathbf{y}_t^\mathrm{fp}]$ and $\Sigma^{yy} = \mathrm{Cov}[\mathbf{y}_t^\mathrm{fp}]$ in section 2.2.3, we estimate **H**_*w*_ and Λ_0_ from them by definition, as:

\begin{align*} \begin{aligned} \mathbf{H}_w&amp; = \mathrm{Cov}\left[\mathbf{w}^\mathrm{f},\mathbf{w}^\mathrm{p}\right] = \mathrm{Cov}\left[\left(\begin{array}{c}\mathbf{z}^\mathrm{f}_{t}\\ \hline \mathbf{y}^\mathrm{f}_{t}\end{array}\right),\left(\begin{array}{c}\mathbf{z}^\mathrm{p}_{t}\\ \hline \mathbf{y}^\mathrm{p}_{t}\end{array}\right)\right]\\ &amp; = \left(\begin{array}{c|c} \mathbf{H}_{w}^{zz}&amp; \mathbf{H}_{w}^{zy}\\ \hline \mathbf{H}_{w}^{yz}&amp; \mathbf{H}_{w}^{yy} \end{array}\right),\\ \end{aligned} \end{align*}

with

\begin{align*} \left\{\begin{aligned} \mathbf{H}_{w}^{zz}&amp; = \Sigma^{zz}\left(1:h_zn_z,h_zn_z+1:2h_zn_z\right),\\ \mathbf{H}_{w}^{zy}&amp; = \Sigma^{zy}\left(1:h_zn_z,h_yn_y+1:2h_yn_y\right),\\ \mathbf{H}_{w}^{yz}&amp; = \Sigma^{zy}\left(h_zn_z+1:2h_zn_z,1:h_yn_y\right)^{^{\prime}},\\ \mathbf{H}_{w}^{yy}&amp; = \Sigma^{yy}\left(1:h_yn_y,h_yn_y+1:2h_yn_y\right), \end{aligned}\right. \end{align*}

and

\begin{align*} \Lambda_{0}&amp; = \mathrm{Cov}\left[\left(\begin{array}{c}\mathbf{z}_{t}\\ \mathbf{y}_{t}\end{array}\right)\right]\\[4pt] &amp; = \left(\begin{array}{c|c} \Lambda_{0}^{zz}&amp; \Lambda_{0}^{zy}\\ \hline \Lambda_{0}^{yz}&amp; \Lambda_{0}^{yy} \end{array}\right)\\[4pt] &amp; = \left(\begin{array}{c|c} \Sigma^{zz}\left(1:n_z,1:n_z\right)&amp; \Sigma^{zy}\left(1:n_z,1:n_y\right)\\ \hline \Sigma^{zy}\left(1:n_z,1:n_y\right)^{^{\prime}}&amp; \Sigma^{yy}\left(1:n_y,1:n_y\right) \end{array}\right), \end{align*}

where : is the standard MATLAB operation for selecting some or all of rows and columns.

## Appendix D Derivation of $\Lambda_{k}^{yy}$, $\Lambda_{k}^{zz}$, $\Lambda_{k}^{yz}$, $\Lambda_{k}^{zy}$ in equation (19) in terms of model parameters

Given that noises **q**_*t*_ and **r**_*t*_ are white (equations (1) and (2)), we know that $\mathbf{q}_{t+i}$ and $\mathbf{r}_{t+i}$ are orthogonal to **y**_*t*_ and **z**_*t*_ for all all positive integer *i*’s. Thus, we have:

\begin{align*} \Lambda_{k}^{yy}&amp;\triangleq\mathrm{Cov}\left[\mathbf{y}_{t+k},\mathbf{y}_{t}\right]\\ &amp; = \mathrm{Cov}\left[\mathbf{C}_{y}\mathbf{A}^{k-1}\mathbf{x}_{t+1},\mathbf{y}_{t}\right]\\ &amp; = \mathbf{C}_{y}\mathbf{A}^{k-1}\mathbf{G}_{y}, \end{align*}

\begin{align*} \Lambda_{k}^{zz}&amp;\triangleq\mathrm{Cov}\left[\mathbf{z}_{t+k},\mathbf{z}_{t}\right]\\ &amp; = \mathrm{Cov}\left[\mathbf{C}_{z}\mathbf{A}^{k-1}\mathbf{x}_{t+1},\mathbf{z}_{t}\right]\\ &amp; = \mathbf{C}_{z}\mathbf{A}^{k-1}\mathbf{G}_{z}, \end{align*}

\begin{align*} \Lambda_{k}^{zy}&amp;\triangleq\mathrm{Cov}\left[\mathbf{z}_{t+k},\mathbf{y}_{t}\right]\\ &amp; = \mathrm{Cov}\left[\mathbf{C}_{z}\mathbf{A}^{k-1}\mathbf{x}_{t+1},\mathbf{y}_{t}\right]\\ &amp; = \mathbf{C}_{z}\mathbf{A}^{k-1}\mathbf{G}_{y}, \end{align*}

\begin{align*} \Lambda_{k}^{yz}&amp;\triangleq\mathrm{Cov}\left[\mathbf{y}_{t+k},\mathbf{z}_{t}\right]\\ &amp; = \mathrm{Cov}\left[\mathbf{C}_{y}\mathbf{A}^{k-1}\mathbf{x}_{t+1},\mathbf{z}_{t}\right]\\ &amp; = \mathbf{C}_{y}\mathbf{A}^{k-1}\mathbf{G}_{z}. \end{align*}

## Appendix E Derivation of the relation of noise covariance parameters to other model parameters

Based on the multiscale dynamical model described in equation set (2), we can write the following equations for $\mathrm{Cov}[\mathbf{x}_t]$, $\mathrm{Cov}[\left(\begin{array}{c}\mathbf{z}_{t}\\ \mathbf{y}_{t}\end{array}\right)]$ and $\mathrm{Cov}[\mathbf{x}_{t+1},\left(\begin{array}{c}\mathbf{z}_{t}\\ \mathbf{y}_{t}\end{array}\right)]$:

\begin{align*} \left\{ \begin{array}{@{}l} \mathrm{Cov}\left[\mathbf{x}_t\right]\triangleq\Sigma_x = \mathbf{A}\Sigma_{x}\mathbf{A}^{^{\prime}}+\mathbf{Q},\\[4pt] \mathrm{Cov}\left[\left(\begin{array}{c}\mathbf{z}_{t}\\ \mathbf{y}_{t}\end{array}\right)\right]\triangleq\Lambda_0 = \left(\begin{array}{c}\mathbf{C}_{z}\\[4pt] \mathbf{C}_{y}\end{array}\right)\Sigma_x\left(\begin{array}{c}\mathbf{C}_{z}\\ \mathbf{C}_{y}\end{array}\right)^{^{\prime}}+\mathbf{R},\\ \mathrm{Cov}\left[\mathbf{x}_{t+1},\left(\begin{array}{c}\mathbf{z}_{t}\\ \mathbf{y}_{t}\end{array}\right)\right]\triangleq\left(\begin{array}{c}\mathbf{G}_z^{^{\prime}}\\\mathbf{G_y^{^{\prime}}}\end{array}\right)^{^{\prime}} = \mathbf{A}\Sigma_x\left(\begin{array}{c}\mathbf{C}_z\\ \mathbf{C}_y\end{array}\right)^{^{\prime}}+\mathbf{S}, \end{array} \right. \end{align*}

where $\mathbf{R}\triangleq\mathrm{Cov}\left[\left(\begin{array}{c}\mathbf{r}_{z,t}\\ \mathbf{r}_{y,t}\end{array}\right)\right] = \left(\begin{array}{cc}\mathbf{R}_z &amp; \mathbf{R}_{zy}\\ \mathbf{R}_{zy}^{^{\prime}} &amp; \mathbf{R}_{y} \end{array}\right)$ and $\mathbf{S}\triangleq\mathrm{Cov}[\mathbf{q}_t,\left(\begin{array}{c}\mathbf{r}_{z,t}\\ \mathbf{r}_{y,t}\end{array}\right)]$. The relations in equation (39) can be easily obtained by rearranging the terms in the above equation set:

\begin{align*} \left\{ \begin{aligned} \mathbf{Q}&amp; = \Sigma_{x}-\mathbf{A}\Sigma_{x}\mathbf{A}^{^{\prime}},\\ \mathbf{R}&amp; = \left(\begin{array}{cc}\mathbf{R}_{z} &amp; \mathbf{R}_{zy}\\ \mathbf{R}_{zy}^{^{\prime}} &amp; \mathbf{R}_{y} \end{array}\right)\\ ~&amp; = \Lambda_{0}-\left(\begin{array}{c} \mathbf{C}_{z}\\ \mathbf{C}_{y}\end{array}\right) \Sigma_{x} \left(\begin{array}{c}\mathbf{C}_{z}\\ \mathbf{C}_{y}\end{array}\right)^{^{\prime}},\\ \mathbf{S}&amp; = \left(\begin{array}{cc} \mathbf{G}_{z}&amp; \mathbf{G}_{y}\end{array}\right)-\mathbf{A}\Sigma_{x} \left(\begin{array}{c}\mathbf{C}_{z}\\ \mathbf{C}_{y}\end{array}\right)^{^{\prime}}. \end{aligned} \right. \end{align*}

## Appendix F Computation of $\Sigma_x$ from model parameters

$\Sigma_x\triangleq \mathrm{Cov}[\mathbf{x}_{t}]$ specifies the covariance of the latent state **x**_*t*_. Given the state transition matrix **A** and the state noise **Q** in equation (1), $\Sigma_x$ can be computed analytically by solving the discrete Lyapunov equation:

\begin{align*} \Sigma_x = \mathbf{A}\Sigma_x\mathbf{A}^{^{\prime}}+\mathbf{Q}. \end{align*}

We use the ‘dlyap’ MATLAB command to solve this equation and find $\Sigma_x$.

## Appendix G Generating **C**_*y*_ and **C**_*z*_ for simulating neural activity in section 2.3

We generate $\mathbf{C_y}\in\mathbb{R}^{n_y\times n_x}$ and $\mathbf{C_z}\in\mathbb{R}^{n_z\times n_x}$ according to the following steps:

(i) We first set the desired contribution of modes as follows:
  (a) If mode *i* is a distinct spiking mode, i.e. only present in spiking activity (and absent in field potential activity), we set $\mathrm{cntrb}_{z,i} = 1$ and $\mathrm{cntrb}_{y,i} = 0$ (equation (41)).
  (b) If mode *i* is a distinct field mode, i.e. only present in field potential activity (and absent in spiking activity), we set $\mathrm{cntrb}_{z,i} = 0$ and $\mathrm{cntrb}_{y,i} = 1$.
  (c) If mode *i* is a shared mode, i.e. present in both modalities, we set $\mathrm{cntrb}_{z,i} = 1$ and $\mathrm{cntrb}_{y,i} = 1$.
(ii) We randomly generate entries of two matrices $\mathbf{C}^{0}_{y}$ and $\mathbf{C}^{0}_{z}$ with elements uniformly distributed in the range $[-1,1]$, whose dimensions are the same as **C**_*y*_ and **C**_*z*_, respectively.
(iii) To adjust the required contribution of each mode in each modality (spiking or field potential activity), i.e. $\mathrm{cntrb}_{z,i}$ and $\mathrm{cntrb}_{y,i}$ in equation (41), we scale the columns corresponding to each mode in $\mathbf{C}^{0}_{y}$ and $\mathbf{C}^{0}_{z}$, accordingly. We denote the scaled matrices as $\mathbf{C}^{1}_{y}$ and $\mathbf{C}^{1}_{z}$. This leads to columns that correspond to distinct spiking (field) modes in $\mathbf{C}^{1}_{y}$ ($\mathbf{C}^{1}_{z}$) being filled with 0.
(iv) To adjust the desired maximum firing rate for each neuron, we scale each row of $\mathbf{C}^{1}_{z}$, such that the upper bound of the analytical $95\%$ confidence interval of the log firing rate, i.e. $\mathrm{diag}(2\mathbf{C}_{z}\Sigma_x\mathbf{C}^{^{\prime}}_{z}+\mathbf{d}_z)$, matches the log of desired maximum firing rates and denote it by $\mathbf{C}^{2}_{z}$.
(v) Since contribution of each mode in the spiking activity may change after the previous step which scales the rows of $\mathbf{C}^{1}_{z}$, we recalculate the normalized contribution of each mode in the spiking activity $\mathrm{nCntrb}_{z,i}$ from $\mathbf{C}^{2}_{z}$. If the recomputed $\mathrm{nCntrb}_{z,i}$ is within an acceptable range of the original required contribution of each mode, we set $\mathbf{C}_{z} = \mathbf{C}^{2}_{z}$ and go to the next step; otherwise, we go back to step (b) to regenerate $\mathbf{C}^{0}_{z}$. We set the acceptable range as $[0.8,1.2]$ times the original required normalized contribution.
(vi) Finally, we scale the whole $\mathbf{C}^{1}_{y}$ such that the sum of contribution of all the modes in the field potential activity is $\frac{n_y}{n_z}$ times that of the spiking activity (log firing rates) and denote it by **C**_*y*_. This step makes sure that the variance of log firing rates and field potential activity that is generated by the dynamical modes is proportional to the number of signals from each modality and the total variance is not dominated by one modality.

## Appendix H Estimating the similarity transform in section 2.3.2

The multiplication of the latent states by any invertible matrix **F**, also known as a similarity transform, gives an equivalent model with the same neural activity **y**_*t*_ and **N**_*t*_ but different basis for the latent state. More precisely, the set of parameters $\mathcal{P} = \{\mathbf{A},\mathbf{C}_{z},\mathbf{C}_{y},\mathbf{G}_{z}, \mathbf{G}_{y},\Lambda_0,\mathbf{d}_z,\Sigma_x\}$ with the latent state **x**_*t*_ will describe the same observations as a transformed model with latent state $\hat{\mathbf{x}}_{t} = \mathbf{F}\mathbf{x}_{t}$ and parameters:

\begin{align*} \hat{\mathcal{P}} = \left\{\mathbf{F}\mathbf{A}\mathbf{F}^{-1},\mathbf{C}_{z}\mathbf{F}^{-1},\mathbf{C}_{y}\mathbf{F}^{-1},\mathbf{F}\mathbf{G}_{z}, \mathbf{F}\mathbf{G}_{y},\Lambda_0,\mathbf{d}_z,\mathbf{F}\Sigma_x\mathbf{F}^{^{\prime}}\right\}. \end{align*}

Thus, to evaluate the learned parameters against the real parameters for simulated models, we need to account for all the equivalent formulations for that simulated model. We address this with the same approach as used in our prior work [23].

To assess whether the learned parameters are close to any equivalent formulation of the true model or not, we first find the similarity transform $\hat{\mathbf{F}}$ that makes the basis of the latent states for the identified model as close as possible to the basis of the latent states for the true model [23]. To do so, we first generate $T = 10\,000\times n_x$ samples of neural activity from the true model. We then extract the predicted latent states using the MSF [43] for both the true and identified models, denoted by $\mathbf{x}^\mathrm{true}_{t+1|t}$ and $\mathbf{x}^\mathrm{id}_{t+1|t}$, respectively. We then find the similarity transform $\hat{\mathbf{F}}$ by minimizing the mean-squared error between the true predicted latent states and the transformed identified latent states, i.e.

\begin{align*} \min_{\mathbf{F}} \left(\sum_{t = 1}^{t = T}\|\mathbf{F}\mathbf{x}^{\mathrm{id}}_{t+1|t}-\mathbf{x}^{\mathrm{true}}_{t+1|t}\|_{2}^{2}\right). \end{align*}

The analytical solution of this least squares problem is $\hat{\mathbf{F}} = \mathbf{X}^{\mathrm{true}}_{\mathrm{pred}}(\mathbf{X}^{\mathrm{id}}_{\mathrm{pred}})^{\dagger}$, where $\mathbf{X}^{\mathrm{true}}_{\mathrm{pred}}$ and $\mathbf{X}^{\mathrm{id}}_{\mathrm{pred}}$ are matrices whose *t*th column contains $\mathbf{x}^{\mathrm{true}}_{t+1|t}$ and $\mathbf{x}^{\mathrm{id}}_{t+1|t}$, respectively. Having computed $\hat{\mathbf{F}}$, we apply it to the identified model parameters (equation (55)) to get an equivalent model in a basis closer to that of the true model. We can then compare the true and the transformed identified model parameters (section 2.3.2).

## Appendix I Computing the error of learning the eigenvalues of **A** (i.e. normalized mode error) in simulations

To compare the eigenvalues of the identified and true **A** matrices by computing the normalized error measure (equation (42)), we first need to find a consistent ordering for the vectors containing the learned and true eigenvalues. This is because all reorderings of the eigenvalues can be thought of as diagonal elements of **A** in different *equivalent* models in canonical form (i.e. with block-diagonal **A** matrices [90]) that can be obtained with similarity transforms from each other (section 2.3.2). To match the ordering of the true and learned eigenvalue vectors, as in prior work [23], among all the possible $n_x!$ permutations of eigenvalue vector of $\mathbf{A}^\mathrm{id}$, we find the closet one to the eigenvalue vector of $\mathbf{A}^\mathrm{true}$ in terms of Euclidean distance. We then use this vector and compare it with the eigenvalue vector of $\mathbf{A}^\mathrm{true}$ by computing the normalized error measure (equation (42)).

## Appendix J Computing the contribution of dynamical modes to behavior, spiking activity, and LFP activity in NHP datasets

Here we explain how to compute the contribution of an identified mode in the NHP dataset to the dynamics of spiking activity, LFP activity and behavior. To compute the contribution of modes to a modality, we need to first transform all the identified model parameters using a similarity transform **E** that makes the identified **A** a real block diagonal matrix (using MATLAB’s bdschur and cdf2rdf commands). To do so, we transform the set of identified model parameters as $\{\mathbf{E}\mathbf{A}\mathbf{E}^{-1},\mathbf{C}_{y}\mathbf{E}^{-1},\mathbf{C}_{z}\mathbf{E}^{-1},\mathbf{E}\mathbf{Q}\mathbf{E}^{^{\prime}},\mathbf{R}_{y},\mathbf{d}_{z}\}$. In what follows, we assume the use of the transformed model parameter when referring to a model parameter. Each block of **A** then corresponds to a dynamical mode, either real or complex. The contribution of mode *i* to the dynamics of a modality can then be defined as the total covariance between the activity in that modality that is generated from mode *i* and the activity in that modality that is generated from all the identified modes (across all neural dimensions from that modality, either log firing rates **z**_*t*_ or field potential activity **y**_*t*_); this is given as:

\begin{align*} \left\{ \begin{array}{@{}l} \mathrm{cntrb}_{z,i}^\mathrm{cov} = \mathrm{trace}\left(\overline{\mathbf{C}}_{z,i}\overline{\overline{\Sigma}}_{x,i}{\mathbf{C}}_{z}^{^{\prime}}\right),\\[6pt] \mathrm{cntrb}_{y,i}^\mathrm{cov} = \mathrm{trace}\left(\overline{\mathbf{C}}_{y,i}\overline{\overline{\Sigma}}_{x,i}{\mathbf{C}}_{y}^{^{\prime}}\right) \end{array}\right. \end{align*}

where $\overline{\overline{\Sigma}}_{x,i}$ is a submatrix of state covariance $\Sigma_{x}$ with rows corresponding to mode *i* and $\overline{\mathbf{C}}_{z,i}$ ($\overline{\mathbf{C}}_{y,i}$) is a submatrix of **C**_*z*_ (**C**_*y*_) with columns corresponding to mode *i*. We then normalize the contribution of mode *i* to the dynamics of a modality by dividing it with the sum of contribution of all the modes and denote it as:

\begin{align*} \left\{ \begin{array}{@{}l} \mathrm{nCntrb}_{z,i}^\mathrm{cov} = \mathrm{trace}\left(\overline{\mathbf{C}}_{z,i}\overline{\overline{\Sigma}}_{x,i}{\mathbf{C}}_{z}^{^{\prime}}\right)/\mathrm{trace}\left({\mathbf{C}}_{z}{\Sigma}_{x}{\mathbf{C}}_{z}^{^{\prime}}\right)\,\\[6pt] \mathrm{nCntrb}_{y,i}^\mathrm{cov} = \mathrm{trace}\left(\overline{\mathbf{C}}_{y,i}\overline{\overline{\Sigma}}_{x,i}{\mathbf{C}}_{y}^{^{\prime}}\right)/\mathrm{trace}\left({\mathbf{C}}_{y}{\Sigma}_{x}{\mathbf{C}}_{y}^{^{\prime}}\right). \end{array}\right. \end{align*}

Note that the sum of the normalized contribution $\mathrm{nCntrb}^{\mathrm{cov}}_{z,i}$ over all the modes is equal to 1, and similarly for $\mathrm{nCntrb}^{\mathrm{cov}}_{y,i}$. We can similarly compute the contribution of a mode to behavior using the learned behavior readout matrix **L** (equation (44)) instead of $\mathbf{C}_{{z}}$ or $\mathbf{C}_{{y}}$ in equation (57). In our NHP data analysis in section 3.2.2, we report these mode contributions to spiking activity, LFP activity, and behavior using $n_x = 14$ in five folds and seven sessions. We then define the behavior predictive mode in each fold as the mode that has the largest normalized contribution to behavior among all the identified modes in that fold (red dots in figure A3).
